# Rolling microswarms along acoustic virtual walls

**DOI:** 10.1038/s41467-022-35078-8

**Published:** 2022-11-29

**Authors:** Zhiyuan Zhang, Alexander Sukhov, Jens Harting, Paolo Malgaretti, Daniel Ahmed

**Affiliations:** 1 Acoustic Robotics Systems Laboratory, Institute of Robotics and Intelligent Systems, Department of Mechanical and Process Engineering, ETH Zurich, Zurich, 8803 Switzerland; 2 Helmholtz Institute Erlangen-Nürnberg for Renewable Energy (IEK-11), Forschungszentrum Jülich, Erlangen, 91058 Germany; 3 Department of Chemical and Biological Engineering and Department of Physics, Friedrich-Alexander-Universität Erlangen-Nürnberg, Nuremberg, 90429 Germany

**Keywords:** Mechanical engineering, Acoustics

## Abstract

Rolling is a ubiquitous transport mode utilized by living organisms and engineered systems. However, rolling at the microscale has been constrained by the requirement of a physical boundary to break the spatial homogeneity of surrounding mediums, which limits its prospects for navigation to locations with no boundaries. Here, in the absence of real boundaries, we show that microswarms can execute rolling along virtual walls in liquids, impelled by a combination of magnetic and acoustic fields. A rotational magnetic field causes individual particles to self-assemble and rotate, while the pressure nodes of an acoustic standing wave field serve as virtual walls. The acoustic radiation force pushes the microswarms towards a virtual wall and provides the reaction force needed to break their fore-aft motion symmetry and induce rolling along arbitrary trajectories. The concept of reconfigurable virtual walls overcomes the fundamental limitation of a physical boundary being required for universal rolling movements.

## Abstract (web summary)

The application of rolling motion to microswarm navigation and cargo delivery has been constrained by the need for a physical boundary to roll along to date. Here, Zhang et al. solve this problem by introducing a reconfigurable virtual wall implemented by a combination of magnetic and acoustic fields.

## Introduction

Classically, rolling is regarded as a type of motion that combines the rotational motion of an object with its translation across a physical boundary. Rolling motion is ubiquitous throughout nature, occurring at scales from the macro to the nano. Tumbleweeds^1^, web-toed salamanders^2^, and golden wheeling spiders^3^ all utilize rolling to disperse seeds or avoid predators. Vampire amoebae^4^ and neutrophils^5^ adopt rolling to propel along cellular or vascular walls. The rolling motion is also commonly used in engineered systems such as trains, automobiles, and robots^6,7^. Being well-established as an effective transport mechanism, rolling is also increasingly studied for the navigation and manipulation of artificial objects at the micro/nano-scale, where viscous forces usually dominate inertial effects^8,9^.

To date, self-assembly microswarms that generally propel by means of a rolling-type motion^10–13^ are becoming attractive since they do not require special prefabricated elements (e.g., sophisticated structures^14^, assembly steps^15^, surface modifications^16^, and so on) and due to their flexible motility, which confers more degrees of freedom for navigation in lab-on-chip microsystems^17^ and complex in vivo vasculature networks^18,19^. However, to convert rotational motion into rolling motion in liquid, existing strategies predominantly rely on a physical boundary (liquid–solid interface) to introduce spatially asymmetrical hydrodynamic interactions^20–22^. Whether involving a smooth or textured surface topography, the strong dependence of these rolling strategies on the presence of a boundary is a fundamental limitation that impairs microswarm maneuverability.

In tandem, a variety of strategies for microswimmer and microswarm motility have been developed, including ones based on chemical gradients^23–27^, bacterial power^28,29^, or external fields such as magnetic^30–37^ and electric^38,39^ fields, as well as energy from light^40–42^, heat^43,44^, and ultrasound^45–58^. Among those, strategies using magnetic and acoustic modalities are intriguing since they allow for the manipulation of micro-objects without contact, they are unscreened (in contrast to electrostatic interactions), unaffected by object opacity (in contrast to optical traps), and they can generate a broad range of forces^59–62^.

**Fig. 1:**
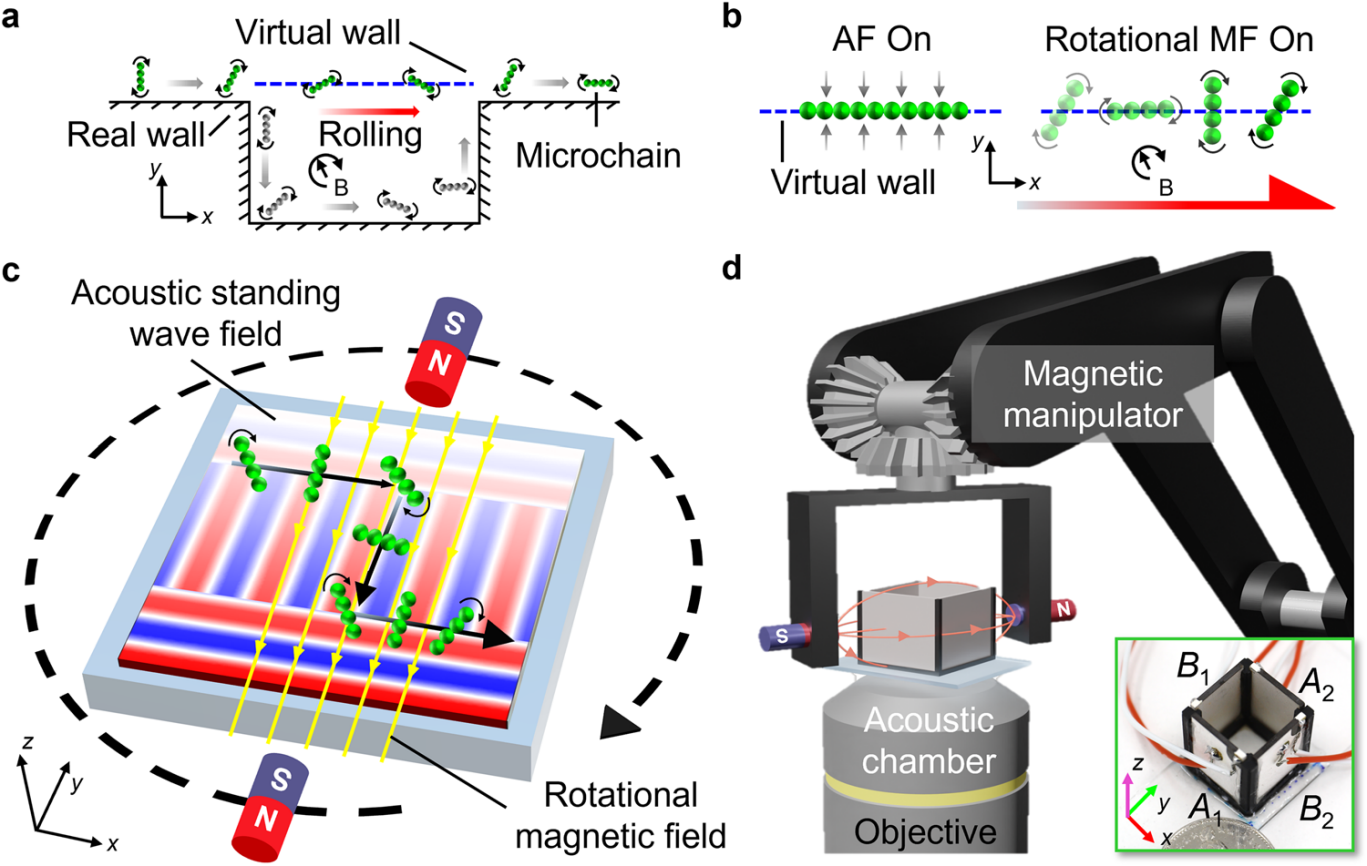
Illustration of chain-shaped microswarms rolling along acoustic virtual walls. **a** Virtual wall concept. To move from left to right by means of rolling in *x*−*y* plane, the gray microchain (conventional methods) has to passively follow the boundary posed by a nearby real wall; in contrast, the green microchain (our strategy) can directly roll forward along the virtual wall. **b** Mechanism of microchain rolling along one-dimensional acoustic virtual walls. (i) Magnetic particles are pushed to the pressure nodal line by the acoustic radiation force as the acoustic standing wave field (AF) is developed. Gray arrows denote the acoustic virtual wall effect from both sides of the acoustic pressure nodal line, which is denoted by the blue dotted line. (ii) Magnetic particles assemble into microchains and achieve rolling motion along the acoustic virtual wall as soon as the rotational magnetic field (MF) is introduced. The curved black arrow indicates the magnetic rotational direction. The red arrow shows the rolling direction. **c** Schematic of microchains rolling along two-dimensional dynamic acoustic virtual walls, realized by switching the orientation of the acoustic standing wave field and the rotational direction of the magnetic field. **d** Schematic of the experimental setup, consisting of an acoustic manipulation chamber and a magnetic manipulation system. The whole setup is mounted on an inverted microscope, and we image the rolling motion using high-speed and high-sensitivity cameras. The inset shows the piezoelectric transducer pairs *A*_1,2_ and *B*_1,2_.

Here, we report a novel strategy, based on a combination of acoustic and magnetic fields, to realize rolling of chain-shaped microswarms (microchains) along a virtual wall in liquid in the absence of real walls. Figure 1a illustrates the virtual wall concept: while a conventional wall-reliant microchain (gray) has to passively follow the real physical boundary in order to break its motion symmetry and roll^63^, our microchain (green) can directly roll left-to-right along a virtual wall distant from the real physical boundary. Within our strategy, when microparticles are introduced into an acoustic standing wave field, the acoustic radiation force (ARF) attracts and aligns them toward the pressure nodal lines that serve as the virtual walls. Then, when a rotational homogeneous magnetic field having its rotational axis perpendicular to the acoustic standing wave field is employed, neighboring magnetic particles self-assemble into multiple microchains and start rotating on account of the magnetic dipole-dipole interactions and the induced magnetic moment^11,64^ (Fig. 1b and Supplementary Movie 1). The distributed and time-varying ARF acting on the rotating microchain effectively breaks its motion symmetry and induces rolling. To further understand and describe this observation, we develop an experiment-supported model, which is consistent with our experimental data and provides significant insights into the rolling motion. Finally, by dynamically building a reconfigurable acoustic virtual wall and switching the rotational direction of the magnetic field, we achieve manipulation of microchains along arbitrary trajectories in liquid while far from real surfaces or boundaries (Fig. 1c).

## Results

### System design

Our experimental setup, shown in Fig. 1d, consists of an acoustic manipulation chamber and a magnetic manipulation system. During experiments, we developed two different acoustic manipulation chambers. The first one is within a confined glass capillary where an acoustic pressure nodal line can be developed at the middle of the capillary by two oppositely placed piezoelectric transducers. Within this chamber, we characterized the rolling behavior of microchains along the one-dimensional (1D) virtual wall. The second one is an open acoustic chamber which comprises a square chassis with four piezoelectric transducers that are indicated as transducer pairs *A*_1,2_ and *B*_1,2_ (Supplementary Fig. 1). Within this second chamber, we performed two-dimensional (2D) dynamic rolling. As the energy of the acoustic field scales quadratically with the excitation voltage (see “Methods” for details), the transducer pair with the higher excitation voltage will dominate the acoustic standing wave field in terms of intensity. Therefore, the orientation (parallel to the *x*-axis or *y*-axis, respectively) of the acoustic virtual walls constituted by the pressure nodal lines can be dynamically switched by tuning the excitation voltage of the originating transducers (Supplementary Fig. 2a to 2c and Movie 2).

The magnetic manipulation system comprises a 5-axis robotic arm and a magnetic end-effector positioned around the acoustic chamber. The end-effector consists of a horseshoe-shaped gripper that uses a pair of permanent magnets (with one north pole points to the other south pole) on its sidearms to establish a homogeneous magnetic field. As the robotic arm executes a programmed rotation, the permanent magnets revolve around the chamber, generating a rotational magnetic field in the horizontal *x*−*y* plane, ${{{{{{{\bf{B}}}}}}}}(t)={B}_{0}\cos \omega t{{{{{{{{\bf{e}}}}}}}}}_{x}+{B}_{0}\sin \omega t{{{{{{{{\bf{e}}}}}}}}}_{y}$, where *B*_0_ is the intensity of the magnetic field, *ω* is the angular frequency, and **e**_*x*_ and **e**_*y*_ are the unit vectors. The calculation based on the Biot-Savart law^65^ and the simulation in COMSOL Multiphysics of the magnetic intensity (see “Methods” for details) show that the magnetic field between a pair of permanent magnets is unidirectional. The magnetic field gradient is close to zero in the range of −1000 μm to +1000 μm, which is three orders of magnitude larger than the diameter (1.63 μm) of the magnetic particles used (Supplementary Fig. 2d to 2f). Control experiments have shown that in a static magnetic field, while the microchains gradually increase in length with time, no noticeable motion is observed (Supplementary Fig. 3). Accordingly, the microchains in the central region of the acoustic chamber experience a nearly homogeneous magnetic field, and hence the magnetic field gradient does not contribute to the displacement of microchains.

### Rolling motion

**Fig. 2:**
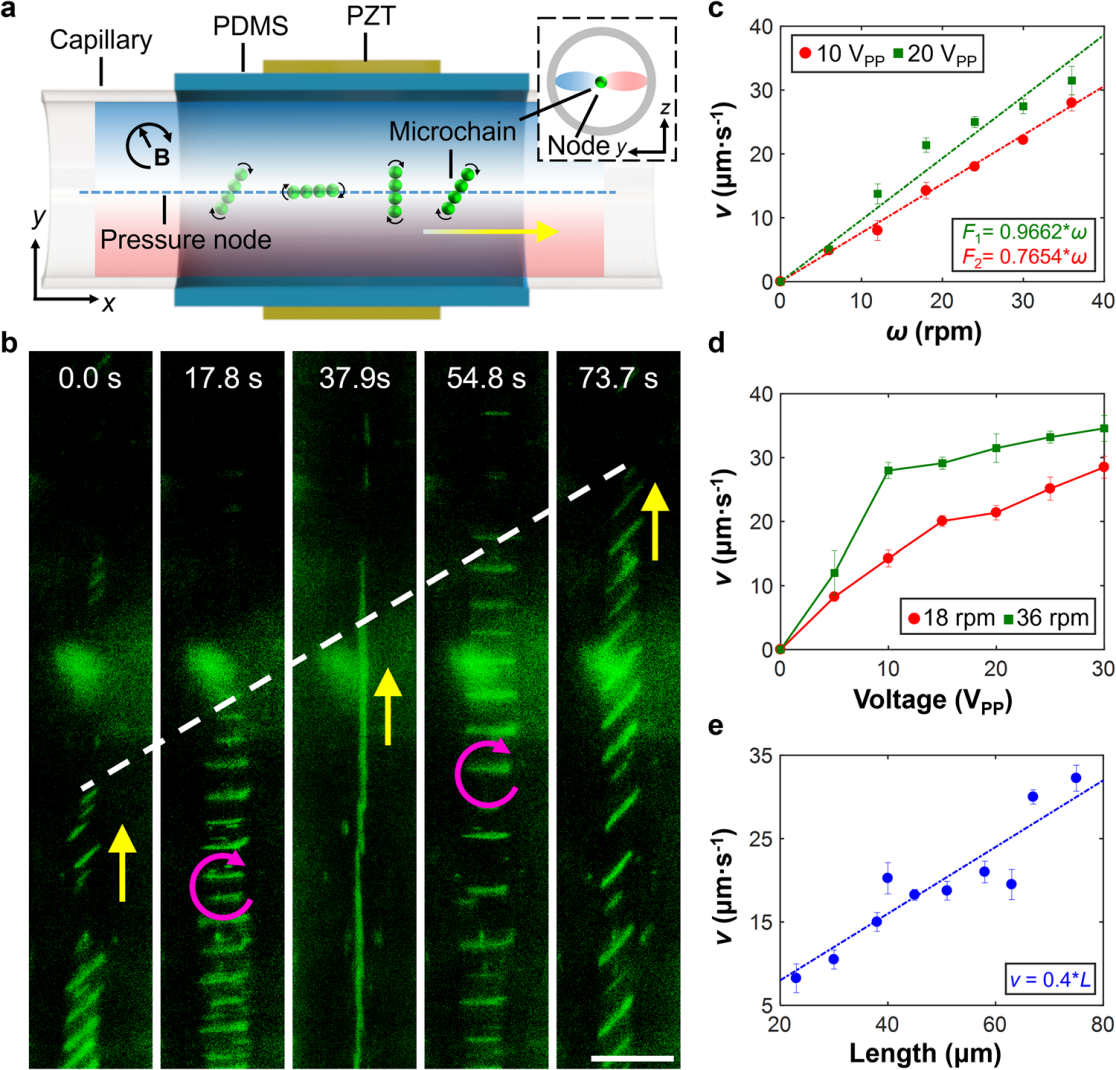
Characterization of microchains rolling along the single acoustic virtual wall in a confined glass capillary. **a** Schematic of the experimental setup. The capillary is held by a polydimethylsiloxane (PDMS) polymer substrate with two piezoelectric transducers (PZT) attached on the substrate’s lateral surfaces. The inset illustrates a cross-sectional view of the glass capillary with the microchain trapped at the pressure node of an acoustic standing wave field. **b** Image sequence of microchains rolling from bottom to top. The pink curved arrow, yellow arrow, and white dotted line respectively denote the clockwise magnetic rotational direction, net translational direction and displacement. Scale bar, 100 μm. **c** Plot characterizing the translational velocity of a single microchain (length 65.63 μm) versus magnetic rotational velocity at selected acoustic voltages. The dotted lines are the corresponding linear fits. **d** Plot characterizing the translational velocity of a single microchain (length 65.63 μm) versus acoustic voltage at different magnetic rotational velocities. **e** Plot of the translational velocity versus microchain length under an acoustic excitation voltage and frequency of 20 V_PP_ and 2.02 MHz, respectively. The magnetic rotational velocity and the magnetic intensity were 24 rpm and 15 mT, respectively. These fittings were performed with the linear function model, *y* = *ax*. Each data point represents the average translational velocity analyzed from 3-5 microchains (Source Data). Error bars represent the standard deviation (s.d.) of data.

To characterize the rolling motion of microchains in the combined acoustic and magnetic field, a set of 1D manipulation experiments with a variety of excitation parameters (namely varying the excitation voltage of the acoustic field and the rotational velocity of the magnetic field) has been performed in a narrow, closed glass capillary with a circular cross-section, as illustrated in Fig. 2a. In the combined presence of acoustic and magnetic fields, microchains exhibited rolling along the acoustic virtual wall (Fig. 2b). During experiments, we observed three rows of rolling microchains in the capillary: one on each side rolling along the capillary boundary, and the third in the middle rolling along the virtual wall (Supplementary Fig. 4). Since all three rows of microchains can be clearly observed under a single microscope focus setting, the virtual wall is implied to be developed in the middle, far from the bottom boundary of the circular capillary. Therefore, experiments in the capillary confirm realization of rolling in the absence of real walls. We then tracked and analyzed a single microchain (with the length of 65.63 μm). Fig. 2c demonstrates the translational velocity of a microchain against the magnetic rotational velocity at excitation acoustic voltages of 10 (red) and 20 (green) V_PP_ (peak-to-peak voltage). As we increase the rotational velocity of the magnetic field, the translational velocity of the microchain increases, revealing a linear relationship, *v* ∝ *ω*. The translational velocity also increases with acoustic voltage, which can be attributed to a higher ARF acting on the microchain (Fig. 2d). In addition, the translational velocity is proportional to the length of the microchains for a fixed acoustic voltage and magnetic rotational velocity as well (Fig. 2e).

### Bidirectional manipulation

**Fig. 3:**
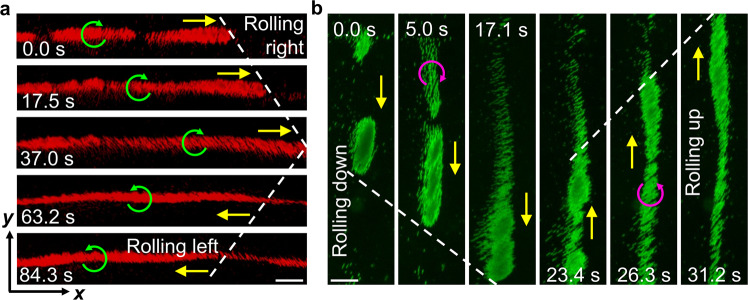
Bidirectional rolling of microchains along the acoustic virtual wall. **a** Bidirectional rolling motion (the direction is indicated by a yellow arrow) along the *x*-axial acoustic virtual wall upon flipping the rotational direction of the magnetic field (green curved arrow). The white dotted line denotes the net displacement. **b** Bidirectional rolling motion along the *y*-axial acoustic virtual wall. The pink curved arrows denote the magnetic rotational direction. For both panels, the acoustic excitation voltage and frequency were 20 V_PP_ and 1.55 MHz, respectively. The magnetic intensity was 21 mT. The magnetic rotational velocity as depicted in panel (**a**) and (**b**) was 24 rpm and 30 rpm, respectively. Scale bar, 100 μm.

To demonstrate the flexibility and stability of our manipulation strategy, we tested the bidirectional rolling of multiple microchains along 1D acoustic virtual walls in the open acoustic chamber (Supplementary Movie 3). In particular, we checked that the direction of rolling can be reverted immediately upon flipping the rotational direction of the magnetic field, as shown in Fig. 3a. The rotational angular velocity of microchains precisely follows the rotational frequency of the magnetic field during multiple rolling cycles (Supplementary Fig. 5). Then, the rolling microchains come to a stop instantaneously by switching off the rotation of the magnetic field. Upon switching the orientation of the acoustic standing wave field with 90 degree, the microchains move in the orthogonal direction (Fig. 3b). In addition, Fig. 3 shows that when multiple microchains are present they may cluster and, within the same cluster, they have different velocities. This can be due to the dispersive nature of microchain properties such as the length, shape, and magnetic moment, which further affects the interaction of microchains with the combined field and surrounding liquid. Concerning the nature of the external fields, the translational velocity of microchains in the open acoustic chamber was also found to increase with increasing magnetic rotational velocity, stronger magnetic intensity, higher acoustic voltage, as well as higher acoustic frequency (Supplementary Fig. 6). Furthermore, microchains demonstrated synchronous rolling along the acoustic virtual walls in multiple suspended planes in the open acoustic chamber suggesting that the chamber’s bottom substrate does not contribute to rolling along acoustic virtual walls (Supplementary Fig. 7 and Movie 4).

### Rolling mechanism and modeling

**Fig. 4:**
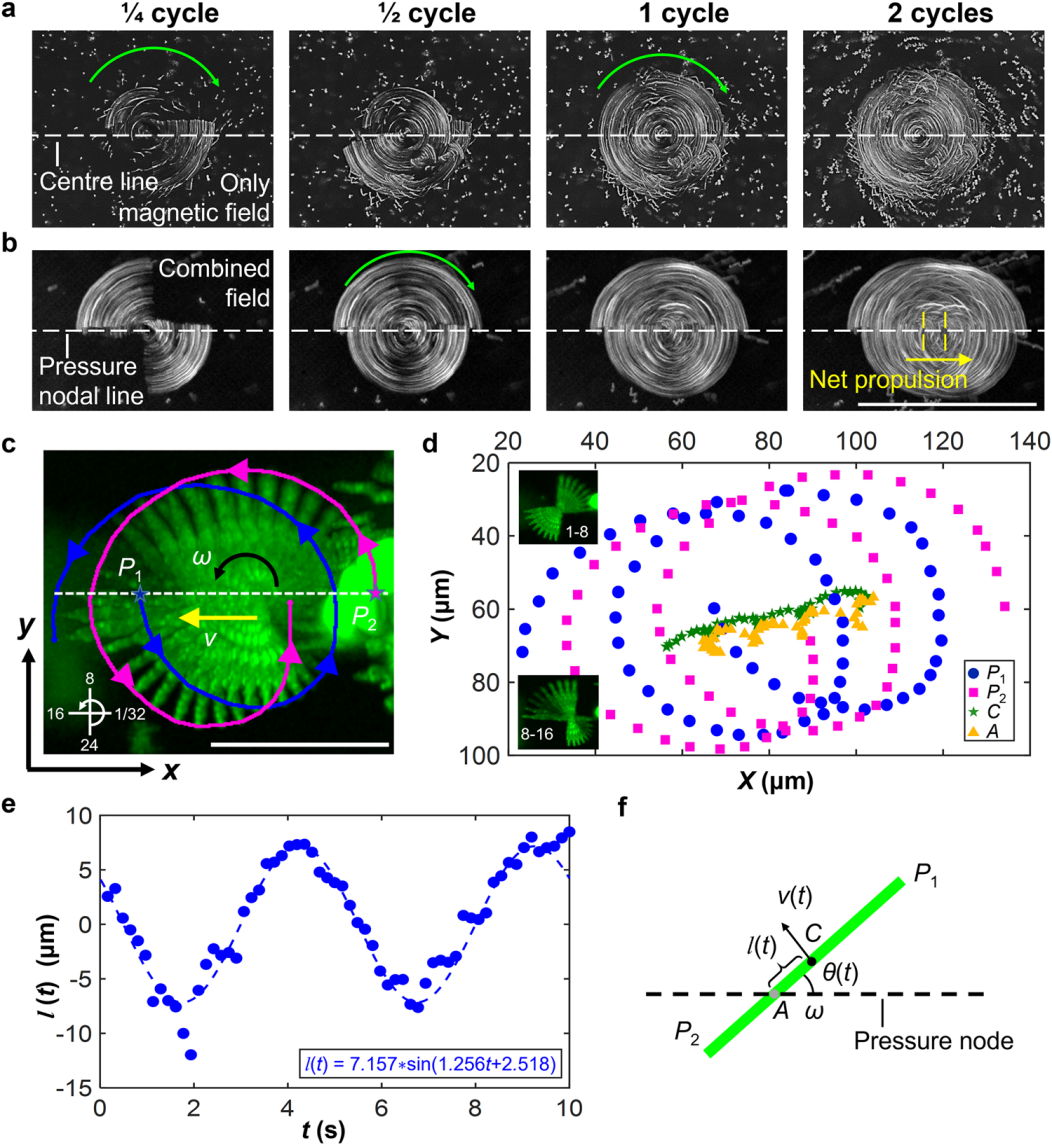
Mechanism of rolling motion along the acoustic virtual wall. **a** Rotation of a microchain in the absence of an acoustic field. The 1/4-cycle superimposed subfigure shows off-center rotation of the microchain, while the 2-cycle subfigure shows its final rotational profile to be like a circle. No noticeable motion is observed. **b** Rotation of a microchain in the presence of a one-dimensional acoustic standing wave field. The microchain exhibits off-center rotation and rolling along the acoustic pressure nodal line. The white dotted lines of (**a**) and (**b**) respectively denote the rotational centerline and the acoustic pressure nodal line. The green curved arrow denotes the rotational direction. The yellow arrow and dotted line denote the rolling direction and the net displacement, respectively. Scale bar, 50 μm. **c** Superimposed time-lapse images of a microchain undergoing one rotational cycle. The acoustic excitation voltage and frequency were 20 V_PP_ and 1.55 MHz, respectively. The magnetic rotational direction was counterclockwise, and the magnetic rotational velocity and intensity were 12 rpm and 21mT, respectively. Blue and pink curves respectively denote the tracked trajectories of the two endpoints, *P*_1_ and *P*_2_. The white dotted line denotes the acoustic pressure nodal line. The axis in the lower left corner denotes the rotational direction. The number refers to the overlapped frames that has the same time interval, 0.1587 s, from 1 to 32. Scale bar, 50 μm. **d** Trajectories of the microchain’s two endpoints (*P*_1_ and *P*_2_), geometric center (*C*), and rotational center (*A*) (Source data). **e** Plot of the variable distance between the geometric and rotational centers of the microchain against rotational time (Source data). The period of one rotational cycle was 5 s. The off-center distance was obtained by subtracting the distance ∣*P*_1_*C*∣ from ∣*P*_1_*A*∣ (Supplementary Fig. 9). Fitting yielded a first-order sine function of $l(t)=7.157*\sin (1.256t+2.518)$. **f** Schematic of the experiment-supported theoretical model. The green rod denotes the self-assembly microchain.

To gain better insight into this rolling motion, we investigated the behavior of microchains in a rotational magnetic field both without and with an acoustic standing wave field. The results show that in the absence of any acoustic wave fields, most of the microchains started symmetrical rotation around their geometric center upon switching on the rotational magnetic field. Although the rotation center of some microchains deviated from the geometric center, the microchains exhibited no net displacement. Figure 4a shows that the final rotational profile of a microchain resembled a circle, implying the motion symmetry was not broken, In contrast, within the presence of a 1D acoustic standing wave field, the rotating microchain exhibited not only an off-center rotation but also a net displacement along the pressure nodal line (Fig. 4b and Supplementary Fig. 8). Control experiments suggest that the rotational magnetic field alone cannot break the time reversibility to induce rolling.

We then tracked and analyzed a single microchain rotating in the combined acoustic and magnetic field with a constant magnetic rotational velocity of 12 rpm (i.e., a rotational period of 5 s) (Supplementary Fig. 9 and Movie 5). The image superposition, Fig. 4c, shows that the microchain has different angular spacings, with values decreasing in frames 1–8 (when the microchain rotates to become perpendicular to the acoustic pressure nodal line) and then increasing in frames 8–16 (when the microchain rotates to become parallel to the acoustic pressure nodal line). Meanwhile, the tracked trajectories and velocities of the microchain’s two endpoints (indicated as *P*_1_ and *P*_2_) show that in a single rotational cycle, the microchain first decelerates and then accelerates (Fig. 4d and Supplementary Fig. 10). Because the ARF acting on the microchain always points to the pressure nodal line, its effective magnitude varies according to the orientation of the microchain (Supplementary Fig. 11). We then calculated the trajectories of the geometric center (indicated as *C*) and rotational center (indicated as *A*) of the microchain (Fig. 4d), and they demonstrate that the distance *l* between the geometric and rotational centers varies across the rotational cycle, with a sinusoidal dependence on rotational time (Fig. 4e). When the long axis of the microchain is parallel to the virtual wall, the center of rotation nearly overlaps with the geometric center; whereas when the long axis becomes perpendicular to the virtual wall, the distance goes to its maximum value. In contrast, the tracking and analysis of a single microchain rotating in the rotational magnetic field without the acoustic standing wave field reveals no apparent rhythmic offset between the geometric and rotational centers (Supplementary Fig. 12). This finding suggests that the action of the time-varying ARF on the microchain is critical for its net displacement.

With this in mind, we developed a theoretical model to capture the net displacement of the rolling microchains along the acoustic virtual wall. In the model, the self-assembly microchain is regarded as a uniform rigid body, as shown in Fig. 4f. According to our previous study^19^, thermal agitation or Brownian motion cannot disrupt the formation of microchains, and the thermal contribution to the viscous force is negligible. We also remark that due to the small size of the microrods and their relatively small velocity, both the inertia of the particle (overdamped regime) as well as the inertia of the fluid (with a low Reynolds number) can be disregarded (Supplementary Note 1). As suggested by experimental data, we regard the distance between the center of geometry and the center of rotation as a function of time, *l*(*t*). Accordingly, the time-averaged net displacement of the geometric center of the microchain, *δ*, can be obtained by integrating the product of the rotational time interval, *dt*, and the linear velocity component in the translational direction within the rotational period, *T*, as

1 $$\delta=\omega \int\nolimits_{0}^{T}\sin (\omega t)l(t){{{{{{{\rm{d}}}}}}}}t,$$

where *ω* = 2*π*/*T* is the angular velocity of the rotational magnetic field. By expressing *l*(*t*) via its Fourier expansion, we obtain

2 $$l(t)={a}_{0}+\mathop{\sum }\limits_{i=1}^{\infty }\left[{a}_{i}\cos (i\omega t)+{b}_{i}\sin (i\omega t)\right],$$

where *a*_*i*_ and *b*_*i*_ are the Fourier parameters. Based on the orthogonality of trigonometric functions for integration, we obtain

3 $$\delta=\omega {b}_{1}\int\nolimits_{0}^{T}\sin (\omega t)\sin (\omega t){{{{{{{\rm{d}}}}}}}}t=\omega {b}_{1}\frac{T}{2}=\pi {b}_{1},$$

4 $$v=\frac{\delta }{T}=\frac{{b}_{1}}{2}\omega,$$

where *b*_1_ is the first item of *b*_*i*_, ${b}_{1}=\frac{2}{T}\int\nolimits_{0}^{T}l(t)\sin (\omega t){{{{{{{\rm{d}}}}}}}}t$, and *v* is the time-averaged translational velocity. Over multiple rotational cycles, *n*, the total net displacement is *n* ⋅ *δ*.

Within the current model, the time-varying acoustic radiation force on the microchains is accounted for by the time-dependent off-center distance *l*(*t*). The model suggests that in the absence of a rotational magnetic field, the angular velocity, *ω* = 0, and no rolling occurs. Likewise, in the absence of the acoustic standing wave field, but with the rotational magnetic field on, *l*, remains constant in time and hence *b*_1_ = 0, leading no rolling motion. Therefore, a microchain undergoes rolling motion only when both rotational magnetic and acoustic standing wave fields are applied. To verify the model, we extracted *l*(*t*) from the experimental data and fitted it with $l(t)=\tilde{l}\sin (\tilde{\omega }t)$, as shown in Fig. 4e. Using the fitting parameters, $\tilde{l}$ and $\tilde{\omega }$, (3), and (4) the time-averaged displacement *δ* and velocity *v* of the tracked microchain (Fig. 4c) over a single rotational cycle read *δ*_*theory*_ = 22.49 μm and *v*_*theory*_ = 4.49 μm ⋅ s^−1^ which are consistent with the tracked experimental data of *δ*_*exp*_ = 22.1 μm and *v*_*exp*_ = 4.4 μm ⋅ s^−1^. The model was then validated by testing multiple rolling microchains under an array of experimental conditions (Supplementary Fig. 13 and Table 1). The error between model calculations and experimental data are <2.5 μm and <1.5 μm ⋅ s^−1^, which are tolerable given the microchain length.

Since the sign (plus or minus) of *v* is synchronized with the sign of *ω*, the model suggests that the translational direction of microchains can be flipped and controlled by the magnetic rotational direction. Based on our model, Fig. 2c is expected that increasing the magnetic rotational velocity causes an increase in the microswarm’s angular velocity. Subsequently, a higher translational velocity is achieved, as described in (4). Meanwhile, experimental results demonstrate that the maximum value of *l*(*t*) (which is approximately equal to *b*_1_ while fitting) increases with the acoustic voltage and microchain length due to a higher ARF (Supplementary Fig. 14). Therefore, the increasing translational velocity, shown in Fig. 2d and Fig. 2e, are achieved because of a larger *b*_1_, which in the model can cause an increased *v*. The model, however, has no analytic expression for the parameters characteristic of the combined field (such as acoustic voltage, acoustic frequency, and magnetic intensity), nor for the microchain length and thickness. A future work will examine the coupling effect of the combined field.

### Two-dimensional dynamic manipulation

**Fig. 5:**
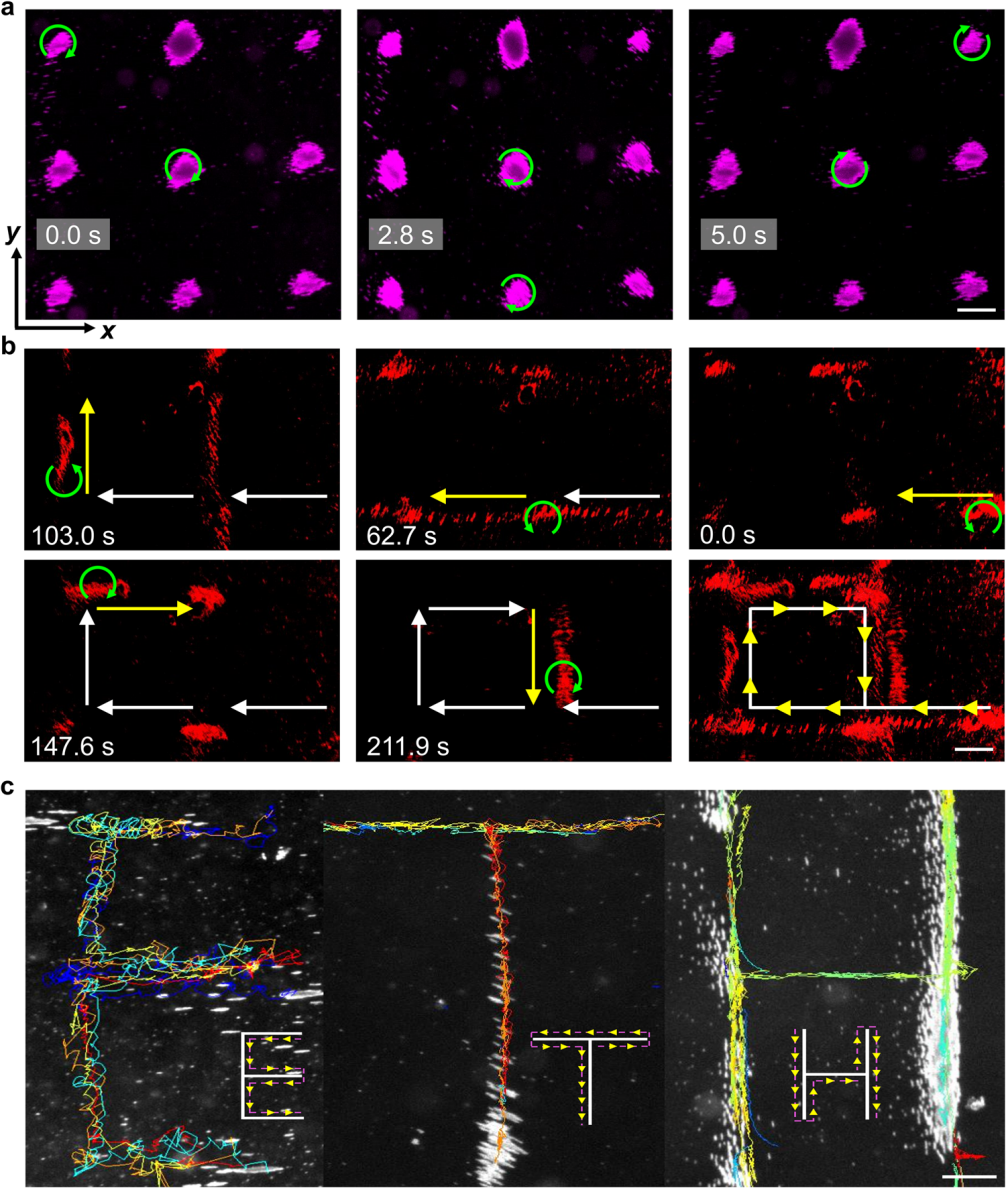
Two-dimensional dynamic rolling manipulation. **a** In situ rotation of microchains at two-dimensional acoustic pressure nodal points. **b** Rolling of microchains along a predesigned square path. The last subfigure is a superimposed time-lapse image showing the full path. The green curved arrow, yellow arrow, and white arrow respectively denote the rotational direction, rolling direction, and historical path. **c** Using reconfigurable acoustic virtual walls to write the word “ETH” with rolling microchains. The acoustic excitation frequency was 1.55 MHz and the magnetic intensity was 21 mT. Inset figures show the rolling direction. Scale bar, 100 μm.

Here, we investigated the capacity of our microchains to perform 2D rolling along acoustic virtual walls. When a 2D acoustic standing wave field is developed in the open acoustic chamber, suspended microchains are aggregated into lattice-like dots and they only rotate at the acoustic pressure nodal points without translation (Fig. 5a). Accordingly, the 2D acoustic standing wave field is employed as a transfer station at each nodal point to realize smooth turns and lessen positional shifts that may occur when directly switching the orientation of the 1D virtual walls.

We then manipulated the microchains to roll along a predesigned square path in the open acoustic chamber by manual operations (Fig. 5b). First, transducer pair *A* was activated at 20 V_PP_, and transducer pair *B* was activated at 1 V_PP_ to build the *x*-axial acoustic virtual wall. Thus, the microchains executed right-to-left rolling in the counterclockwise rotational magnetic field. Later, the excitation voltage of transducer pair *B* was gradually raised to 20 V_PP_ to develop the 2D acoustic standing wave field and stop the microchains at the nodal point. Then, the *y*-axial acoustic virtual wall was built and the microchains exhibited bottom-to-top rolling upon the excitation voltage of transducer pair *A* was gradually reduced to 1 V_PP_. As the microchains approached the next pressure nodal point, we gradually raised the excitation voltage of transducer pair *A* back to 20 V_PP_ and switched the rotational direction of the magnetic field from counterclockwise to clockwise direction. Subsequently, the excitation voltage of the transducer pair *B* was gradually reduced to 1 V_PP_ and the microchains exhibited left-to-right rolling. Similarly, the *y*-axial acoustic virtual wall was then reconstructed again and the microchains finally rolled back to their initial point from top to bottom. Notably, during switching the orientation of virtual walls, we observed that microchains were accelerated and decelerated depending on its relative position to the new pressure nodal line because of the redeveloped acoustic potential (Supplementary Fig. 15). The dexterity of our manipulation strategy is further illustrated in Fig. 5c, where microchains roll along dynamically reconstructed acoustic virtual walls with trajectories that are depicted by the letters “*E*”, “*T*”, and “*H*” (Supplementary Movie 6). It can be expected that more precisely following of the desired trajectory will be achieved by automatically tuning the acoustic voltage through a program-controlled function generator. These demonstrations confirm the multi-particle dynamic manipulation capacity of our strategy and its potential as an efficient transportation solution. It is worth mentioning that during redirection of microchains, the physical position of the experimental setup, i.e., the acoustic manipulation chamber and the magnetic manipulation system, remains constant. We only modulated the excitation voltage of the transducers and the rotational direction of the magnetic field. Since the rotational axis of the magnetic field was fixed, no precise coordination of the magnetic field is required.

## Discussion

In this work, we report a strategy for manipulating chain-shaped microswarms into rolling without reliance on physical boundaries. Under the combination of an acoustic standing wave field and a rotational magnetic field, magnetic particles are trapped and self-assembled into microchains that execute rolling along acoustic pressure nodal lines, which act as virtual walls. The time-varying distance between the geometric and rotational centers of the microchain accounts for the symmetry breaking and induction of rolling. We further demonstrate dexterous rolling of microchains along arbitrary trajectories, realized by dynamically constructing the virtual walls and switching the rotational direction of the magnetic field. In terms of apparatus, we establish the rotational magnetic field using a 3D-printed frame featuring several commercial permanent magnets, which is both easy to construct and portable. Ultimately, the experiment-supported theoretical model allows us to identify key rolling parameters and provides explanations and insights regarding the rolling mechanism.

We compare the rolling velocities of our microswarms to those obtained in other microroller demonstrations. Magnetic microrollers with special features, such as Janus balls, anisotropic shapes, and rods, were found to roll at speeds ranging from 100–600 μm ⋅ s^−1^, and self-assembling microparticle swarms at speeds of 2–50 μm ⋅ s^−1 ^^10–13,20–22,63^. The rolling velocity of our microchains is on the same order of magnitude as that of self-assembling swarms reported in the literature, but is somewhat lower, which could be attributed to the virtual wall not possessing the no-slip property of a physical wall. Furthermore, the maximum microchain translational velocity in this work is restricted by the rotational rate of the robotic arm (0 to 40 rpm) as well as the acoustic excitation voltage (0 to 30 V_PP_). A high-speed motor can be installed between the magnetic manipulation system and the robotic arm to increase the rotational rate (up to the limit of the step-out frequency). Based on our model, increasing acoustic excitation voltage and frequency as well as the dimensions of the microchain should also increase the translational velocity.

As for the step-out frequency of the swarms, we test two distinct self-assembled shapes of magnetic particles, namely chains and discs, at 21 mT with different magnetic rotational frequencies (Supplementary Fig. 16). We observe that the microparticles self-assembled into long chains and can maintain those chains while the rotational frequency is below 5 Hz. At frequencies of 5 Hz and above, the microswarms disassemble into smaller chains. Upon further increasing the frequency (~8 Hz), those smaller chains reassemble into disk-like structures. Based on our experiments and model, the rolling velocity of a longer chain is faster than that of a shorter chain. With the disk-shaped clusters, we do not observe any noticeable displacement; the acoustic radiation force tends to be uniform for a circular shape, and hence the disk-shaped microswarms would only rotate in place. Given these results, we expect that the step-out frequency of the studied microswarms is around 5 Hz.

To realize more flexible modulation of the acoustic virtual walls and rolling in three-dimensional (3D) space, a transducer array^66–68^ can be designed to establish 3D virtual walls and incorporated with a rotational magnetic field having variable rotational axes. However, when it comes to instrumentation and attaining a higher resolution of 3D manipulation, several practical limitations are expected. (1) Since the spacing between acoustic pressure nodal lines is dictated by excitation frequency, higher excitation frequencies allow finer adjustment, i.e., navigation with higher spatial resolution. However, as the frequency goes above 200 MHz, the acoustic waves could be attenuated significantly due to viscous attenuation by the liquid, thus inhibiting robust pattern formation^69^. (2) Visualization and tracking of microswarms in 3D is another challenge that needs to be addressed. A 45-degree prism can be mounted on the imaging system to allow two cameras to simultaneously record the rolling from different perspectives^70^. (3) Since the rotational axis of the magnetic field needs to be perpendicular to the acoustic pressure nodal lines, it is necessary that the rotational axis be adjustable so as to maintain that configuration when developing the acoustic standing wave field in different planes. This can be achieved by using sophisticated 3D Helmholtz coils or commercially available electromagnetic systems that can tune the rotational magnetic axis^71,72^.

As acoustic and magnetic fields can readily extend into the body, we envision rolling along virtual walls to have applications in medicine. Real physical surfaces are readily available in vivo, but most are uneven, plicate, and rife with intermittent tributaries^73^. Conventional rolling approaches that rely on a physical wall have to follow the boundary, and a complex surface topography may impede or halt the translational movement, e.g., by presenting a discontinuous boundary. Maintaining the driving condition across a heterogeneous surface thus requires the actuating parameters (intensities and orientations) to be adjusted in real-time. As such, wall-reliant rolling demands precision, flexibility, and robustness from the swarm structure, operation strategy, and actuating system. By contrast, rolling along an acoustic virtual wall enables following of a direct path without cumbersome fine-tuning of actuation to account for terrain. Thus, our approach provides a viable alternative to conventional propulsion over rugged terrain. Furthermore, the developed microswarms would be promising for in vivo applications in spaces where walls are absent, e.g., the stomach, peritoneal cavity, and bladder. In these bulk-space conditions, conventional wall-reliant rolling^10–13,20–22,63^ will be difficult to execute. Our method is advantageous for such circumstances due to: (1) the microswarms not requiring any contact with surfaces and (2) the acoustic virtual walls being quickly reconstructed and customizable. Our approach could therefore be an effective and exciting strategy for microswarm traversal of bulk spaces. In addition, our rolling strategy will enrich microfluidic lab-on-chip technologies for the actuation, manipulation, and assembly of micro-objects.

## Methods

### Acoustofluidic setup

The square chassis of the 2D open acoustic chamber with dimensions 24 × 24 × 20 mm is fabricated from black photopolymer resin (Standard Black, Formlabs) by 3D printing (Form 3, Formlabs). Four piezoelectric transducers (SM111, STEMINC) are orthogonally glued to the chassis. According to the technical data sheet by the manufacturer, the transducer resonance frequency is 1.5 MHz ± 4%. Subsequently, the acoustic chamber is glued to a thin glass substrate. Two electronic function generators (TG1006, Aim-TTi) are connected to the two transducer pairs to generate the ultrasound waves. For the confined glass capillary chamber, a capillary with the outer diameter of 1.5 mm and the inner diameter of 1mm is embedded into a rectangular PDMS (Polydimethylsiloxane) polymer substrate, which is fabricated using the mold replica technique. And two piezoelectric transducers (resonance frequency 2 MHz ± 4%) are glued to the PDMS lateral surfaces to establish the acoustic standing wave field.

### Magnetic setup

The horseshoe-shaped gripper of the magnetic manipulation system with dimensions 44 × 42 × 10 mm is fabricated from black photopolymer resin by 3D printing. To build the magnetic field, permanent magnets (S-10-05-N52N, NdFeB, Supermagnete) are symmetrically mounted on both sidearms of the gripper. To rotate the magnetic field, the magnetic device is mounted to the end of a 5-axis robotic arm (Dorna 2, Dorna Robotics). In addition, the magnetic intensity can be adjusted by adding external permanent magnets to or removing them from the sidearms (Supplementary Fig. 2f).

### Experimental particles

Commercially available encapsulated magnetic polymer particles having the diameter of 1.63 μm (COMPEL, Bangs Laboratories) are utilized in our experiments. Benefitting from the high susceptibility, these particles react quickly to the external magnetic field. Since, the acoustic excitation frequency of our transducer pairs is around 1.5 MHz corresponding to acoustic wavelength, *λ*, around 1 mm. The radius, *a*, of these particles ensures the validity of the basic assumption, *a* < < *λ*, of the Gor’kov theory.

### Acoustic radiation force

When acoustic standing waves are generated in a liquid containing suspended microparticles, the acoustic radiation force which comprised of primary and secondary radiation forces will arise^74^. The primary radiation force arises from the scattering of the background acoustic standing waves on the particle, whereas the secondary radiation force results from the scattered waves between adjacent particles. Here, we focus on the primary radiation force which plays an important role on aggregating microparticles. Based on the gradient of the Gor’kov potential^75^, the time-averaged primary radiation force acting on a single isolated incompressible particle in a one-dimensional acoustic standing wave field can be expressed as

5 $${F}_{AR}=4\pi \phi (\widetilde{\kappa },\widetilde{\rho })k{a}^{3}{E}_{ac}\sin (2kx),$$

6 $$\phi (\widetilde{\kappa },\widetilde{\rho })=\frac{1}{3}\left[\frac{5\widetilde{\rho }-2}{2\widetilde{\rho }+1}-\widetilde{\kappa }\right],$$

7 $$\widetilde{\rho }=\frac{{\rho }_{s}}{{\rho }_{0}},\widetilde{\kappa }=\frac{{\kappa }_{s}}{{\kappa }_{0}},$$

where *a* is the radius of the microparticle which is considerably smaller than the acoustic wavelength *λ*; *ϕ* is the acoustic contrast factor. The used magnetic particles have a positive contrast factor, which is estimated to be 0.29 (Supplementary Note 2); *k* is the wavenumber of the standing wave with the pressure field *P* = ${P}_{a}\cos (kx)\cos ({\omega }_{a}t)$, *P*_*a*_ denotes the pressure amplitude, *x* determines the position of the particle, *ω*_*a*_ is the propagating frequency; *E*_*ac*_ denotes the acoustic energy; *ρ*_0_, *ρ*_*s*_ denote the density of the liquid and particle, respectively; *κ*_0_, *κ*_*s*_ denote the compressibility of the liquid and particle, respectively^76^.

### Relationship between the acoustic radiation force and excitation voltage

The acoustic energy *E*_*ac*_ can be obtained as

8 $${E}_{ac}=\frac{{P}_{a}^{2}}{4{\rho }_{0}{c}_{0}^{2}}=\frac{1}{4}{\rho }_{0}{v}_{0}^{2},$$

where *v*_0_ is the induced acoustic velocity; *c*_0_ denotes the speed of sound in water, 1500 m ⋅ s^−1^. In study^77^, it is shown that the induced acoustic pressure amplitude *P*_*a*_ is linear to the applied peak-to-peak voltage, V_PP_, of the piezoelectric transducer. Consequently, we obtain the following equation

9 $${F}_{AR}\propto {E}_{ac}\propto {P}_{a}^{2}\propto {V}_{PP}^{2}.$$

### Magnetic intensity of the cylindrical permanent magnet

Based on the Biot-Savart law^65^, the magnetic intensity at any points on the polar axis of the cylindrical permanent magnet can be obtained by

10 $$B(x)=\frac{{B}_{r}}{2}\left[\frac{L+x}{\sqrt{{R}^{2}+{\left(L+x\right)}^{2}}}-\frac{x}{\sqrt{{R}^{2}+{(x)}^{2}}}\right],$$

where *B*_*r*_ is the remanence of the magnet, for the N52N magnetic material, the reference value of remanence is 1.44 T; *L* and *R* is the length and radius of the magnet, respectively; *x* is the distance between the test point and the magnet. In this work, we used a pair of magnets to build the magnetic field. The total distance, *S*, between the magnetic pair is 40 mm. Thus, the magnetic intensity in the range (−20 mm, 20 mm) can be calculated as

11 $$B=B(x)+B(S-x).$$

### Numerical simulation

Finite element numerical simulations are conducted using the commercial COMSOL Multiphysics software (v5.6, Burlington, MA). We perform the acoustic standing wave field simulations with the “Pressure Acoustics, Frequency Domain” module. The inward “Normal Displacement” of the domain boundary is enabled to denote the oscillation from piezoelectric transducers. The simulated acoustic pressure pattern is consistent with our experimental results. For magnetic simulations, the “Magnetic Field, No Current” module is used. The material of the permanent magnet is set as N52 (Sintered NdFeB) and the magnetization model is “Relative permeability”. A square airbox is employed as the calculation domain. All geometrical parameters are set as the experimental setup and the physics-controlled mesh is utilized. The simulated magnetic intensity is consistent with our numerical calculations.

### Imaging and data analysis

The acoustic chamber is mounted on an inverted microscope (Axiovert 200M, ZEISS) equipped with a CCD Monochrome Camera (Photometrics Cool SNAP EZ, Boston Microscopes) and a high-speed camera (Chronos 1.4, Kron Technologies). The angular velocity and translational velocity of microchains are analyzed by video analysis in ImageJ^78^.

### Reporting summary

Further information on research design is available in the Nature Portfolio Reporting Summary linked to this article.

## Supplementary information

Supplementary Information

Description of Additional Supplementary Files

Supplementary Movie 1

Supplementary Movie 2

Supplementary Movie 3

Supplementary Movie 4

Supplementary Movie 5

Supplementary Movie 6

Reporting Summary

## Data Availability

The authors declare that data supporting the findings of this study are available within the paper and its Supplementary Information. Source data are provided with this paper.

## Source data

Source Data
