# Supplementary material for: Rolling microswarms along acoustic virtual walls
Source: Nat Commun. 2022 Nov 29;13:7347. doi: 10.1038/s41467-022-35078-8 (PMC9708833; doi:10.1038/s41467-022-35078-8)
Supplement: Supplementary file 1 — Supplementary Information [file 41467_2022_35078_MOESM1_ESM.pdf]

Supplementary Information for

Rolling Microswarms along Acoustic Virtual Walls

Zhiyuan Zhang, Alexander Sukhov, Jens Harting, Paolo Malgaretti, and Daniel Ahmed

Corresponding Author: Daniel Ahmed

E-mail: dahmed@ethz.ch

This PDF file includes:

Supplementary Notes 1 to 2

Supplementary Figures 1 to 16

Supplementary Table 1

Supplementary References

Supplementary Notes

Supplementary Note 1. Estimation of the Reynolds number of the microchain. The Reynolds number of the microchain, $Re = \rho v_s L/\mu$, was estimated as $Re = 5 \times 10^{-3}$, where $\rho = 1000\ \mathrm{kg \cdot m^{-3}}$, the density of deionized water; $L = 100\ \mu\mathrm{m}$, the maximum characteristic length of the microchain; $v_s = 50\ \mu\mathrm{m \cdot s^{-1}}$, the maximum translational velocity of the microchain; and $\mu = 10^{-3}\ \mathrm{Pa \cdot s}$, the fluid viscosity.

The Reynolds number of the particle at the endpoint of a microchain was estimated as $Re = 2.45 \times 10^{-4}$, where $L = 1.63\ \mu\mathrm{m}$, the diameter of the particle; and $v_s = 150\ \mu\mathrm{m \cdot s^{-1}}$, the maximum linear velocity of the particle.

Supplementary Note 2. Acoustic contrast factor. The acoustic contrast factor ϕ dictates the minimum position of the Gor'kov potential [1]. If the density-related ration $(5\tilde{\rho} - 2)/(2\tilde{\rho} + 1)$ is bigger than the compressibility ratio $\tilde{\kappa}$, ϕ is positive and the microparticle will be pushed towards the pressure node of the wave field. Otherwise, the microparticle will be pushed toward the pressure antinode. The density of the deionized water is $1000\ \mathrm{kg \cdot m^{-3}}$. The compressibility of the deionized water, which equals to $k_0 = 1(\rho_0 c_0^2)$, is estimated to be $4.4 \times 10^{-10}\ \mathrm{Pa^{-1}}$, based on the sound speed in water $c_0 = 1500\ \mathrm{m \cdot s^{-1}}$ [2]. The Commercially available magnetic particles of diameter 1.63 $\mu\mathrm{m}$ (COMPEL, Bangs Laboratories) have the density of $1580\ \mathrm{kg \cdot m^{-3}}$ (provided in the technical data sheet by the manufacturer). The compressibility of the polymer/iron composite, can be obtained by the expression

$$K_s = \frac{1}{V}(V_{Fe}K_{Fe} + V_{PS}K_{PS}), \tag{1}$$

where V_{Fe}, V_{PS} is the volume fraction of iron and polystyrene, which is 2% $\sim$ 6% and <16%, respectively [3]; K_{Fe}, K_{PS} is the compressibility of iron and polystyrene, which is $\sim 5.88 \times 10^{-7}\ \mathrm{Pa^{-1}}$ and $\sim 220 \times 10^{-6}\ \mathrm{Pa^{-1}}$ respectively [4]. Therefore, the acoustic contrast factor ϕ of the magnetic particles is positive and was estimated to be ∼0.29, which suggests that the particles will be pushed to the pressure nodal lines of the one-dimensional acoustic standing wave field.

Supplementary Figures

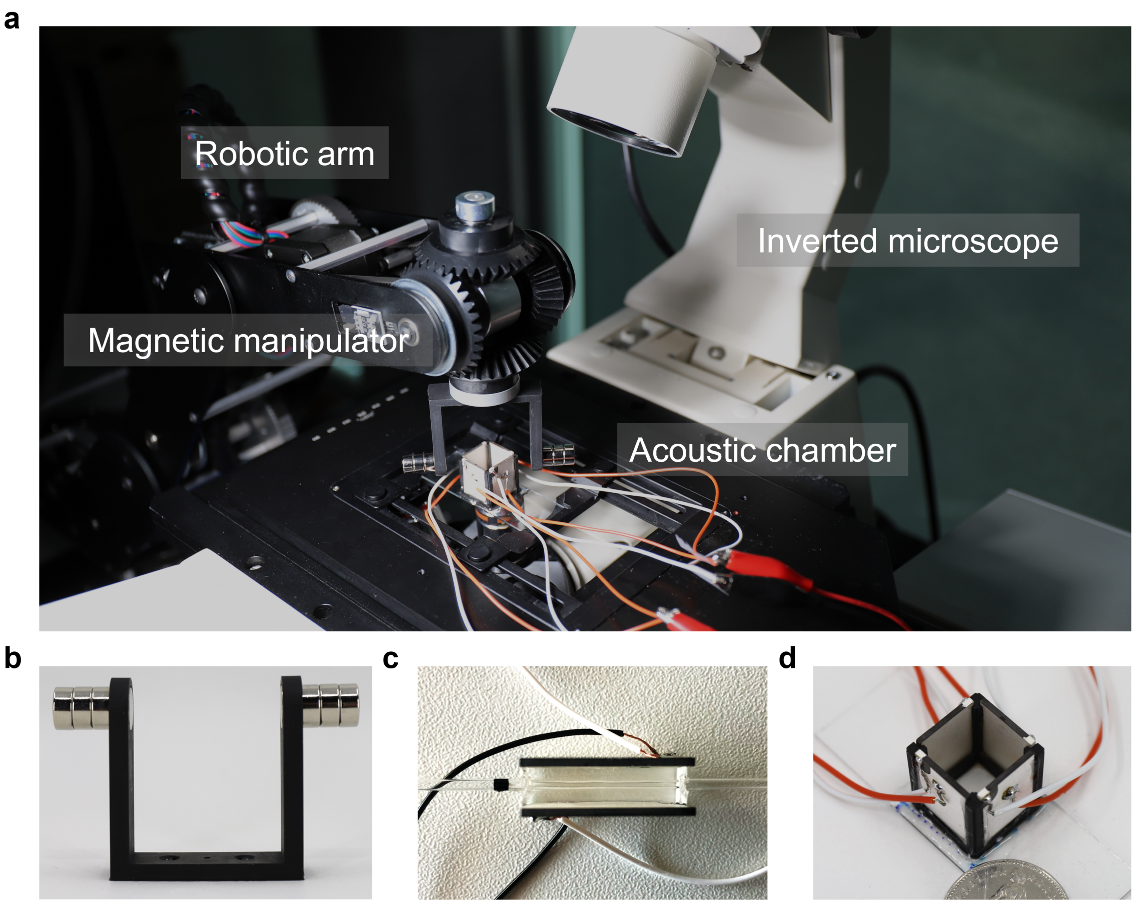

Supplementary Fig. 1. Experimental setup. (**a**) The experimental setup consists of a magnetic manipulation system and an acoustic manipulation chamber. The whole setup is mounted on an inverted microscope and the rolling manipulation is imaged and captured by high-speed and high-sensitivity cameras. (**b**) A horseshoe-shaped gripper to grip permanent magnets. (**c**) A confined glass capillary acoustic chamber consists of two opposing placed piezoelectric transducers. (**d**) An open acoustic chamber consists of four piezoelectric transducers (white) which are orthogonally glued to the square chassis (black).

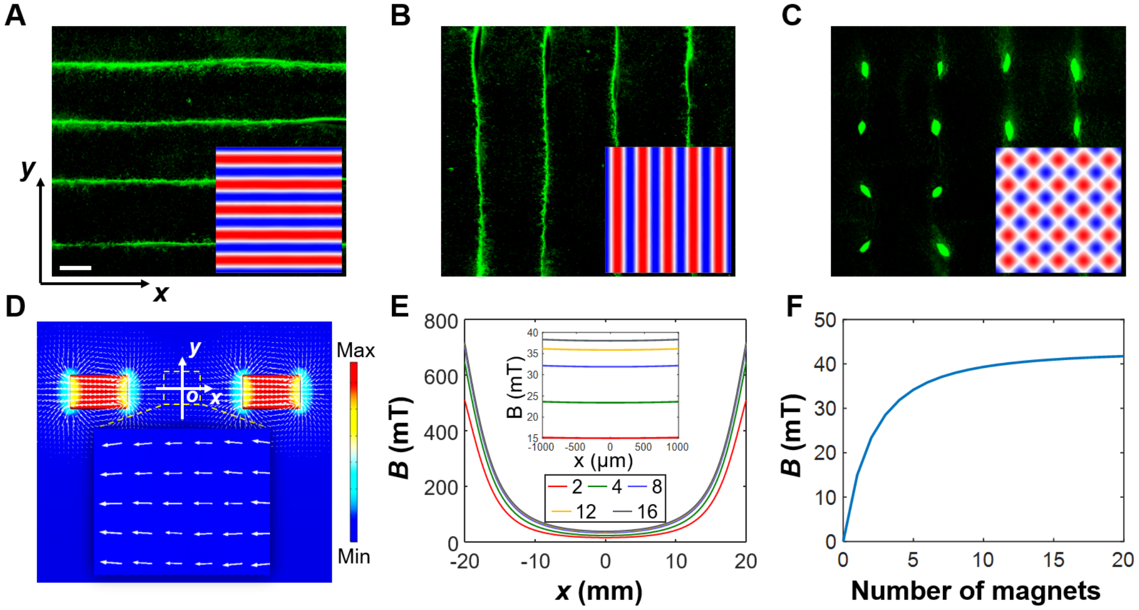

Supplementary Fig. 2. Configuration of experimental setup. (**a** to **c**) The dynamic behavior of the acoustic standing wave field shown by the 1.63 μm magnetic particles. The acoustic excitation voltage and frequency were 20 V_{PP} and 1.55 MHz, respectively. Scale bar, 100 μm. (**a**) When the pair of transducers labeled A_1 and A_2 are activated by a wave signal generated by the corresponding function generator, the x-axial one-dimensional acoustic standing wave field develops in the liquid. (**b**) When the other pair of transducers labeled B_1 and B_2 are activated, the y-axial one-dimensional acoustic standing wave field develops in the liquid. (**c**) When the four transducers are activated simultaneously by similar wave signals, a 2D acoustic standing wave field develops in the liquid. The insets show the corresponding numerical simulations. (**d**) Numerical simulation of the magnetic field. The inset shows the magnetic field in the area of the yellow box. The white arrows show the magnetic direction and the background color denotes the magnetic intensity. (**e**) The plot shows the magnetic intensity along the x-axis (Source Data). The insert figure shows the magnetic intensity in the range from -1000 μm to +1000 μm. The figure legend denotes the number of permanent magnets of the magnetic manipulation system. (**f**) The plot shows the magnetic intensity at the O point versus the number of permanent magnets (Source Data).

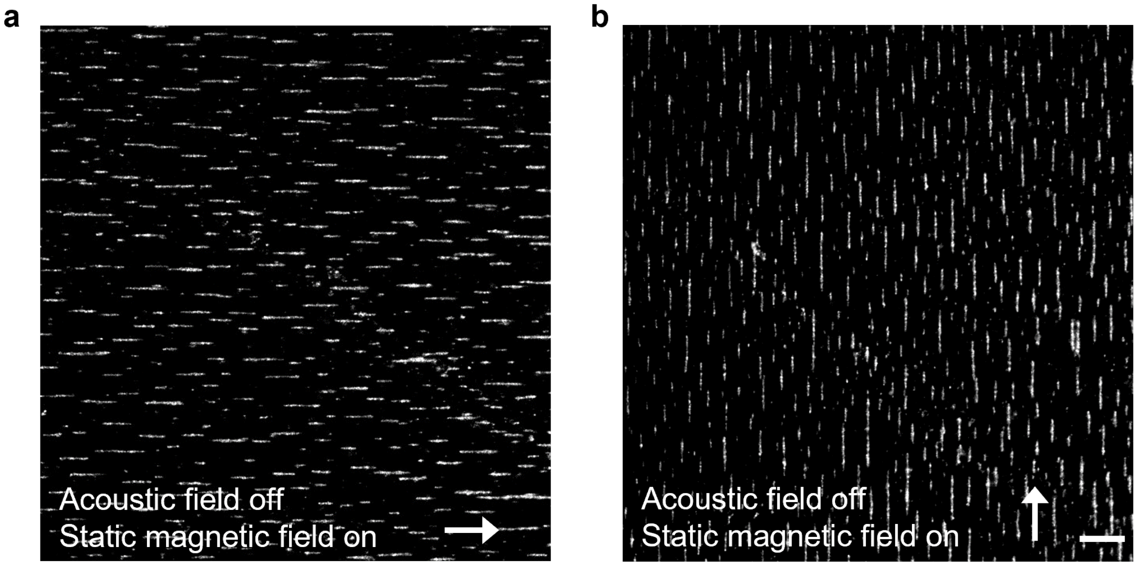

Supplementary Fig. 3. Behavior of microswarms in the static magnetic field without the acoustic field. (**a** to **b**) Chain-shaped microswarms arbitrarily disperse in the liquid and, as time goes on, the length of microchains gradually increases due to the magnetic dipole-dipole interaction between adjacent magnetic particles. No noticeable motion is observed. The magnetic intensity was 21 mT. Scale bar, 100 μm.

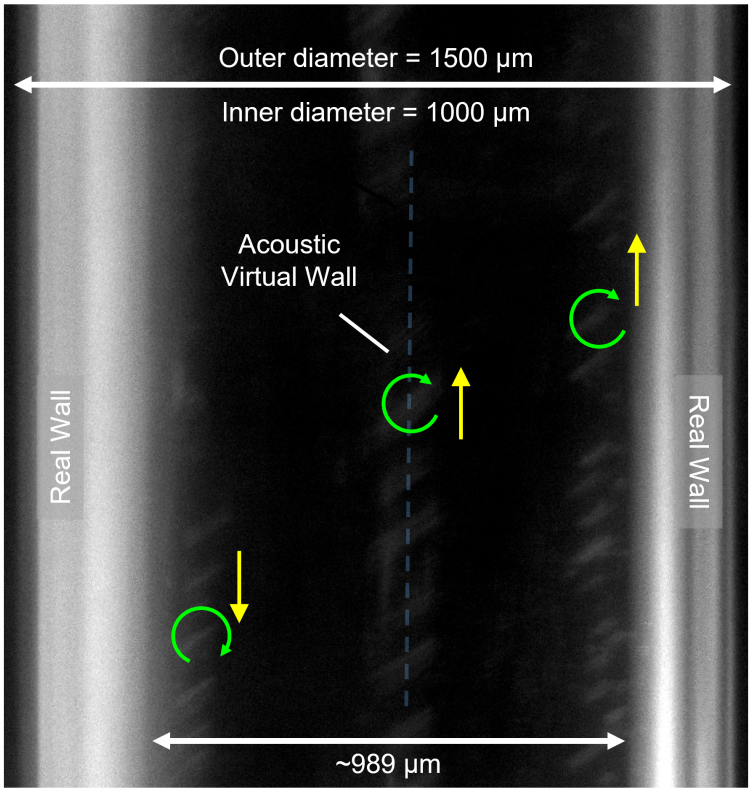

Supplementary Fig. 4. Control experiment in a narrow capillary. Three rows of rolling microchains were observed, among which microchains on both sides rolled along the capillary boundary, the microchains in the middle were identified as rolling along the virtual wall. The outer and inner diameters of the capillary are 1500 μm and 1000 μm, respectively. The distance between the two side rows is nearly 989 μm, implying the virtual wall is developed in the middle and far away from the bottom boundary of the circular capillary. The green curved arrow, the yellow arrow, and the blue dotted line denote the magnetic rotational direction, the rolling direction, and the acoustic virtual wall, respectively. The acoustic excitation voltage and frequency were 20 V_{PP} and 1.55 MHz, respectively. The magnetic rotational direction was counterclockwise, and the magnetic rotational velocity and intensity were 24 rpm and 21 mT, respectively.

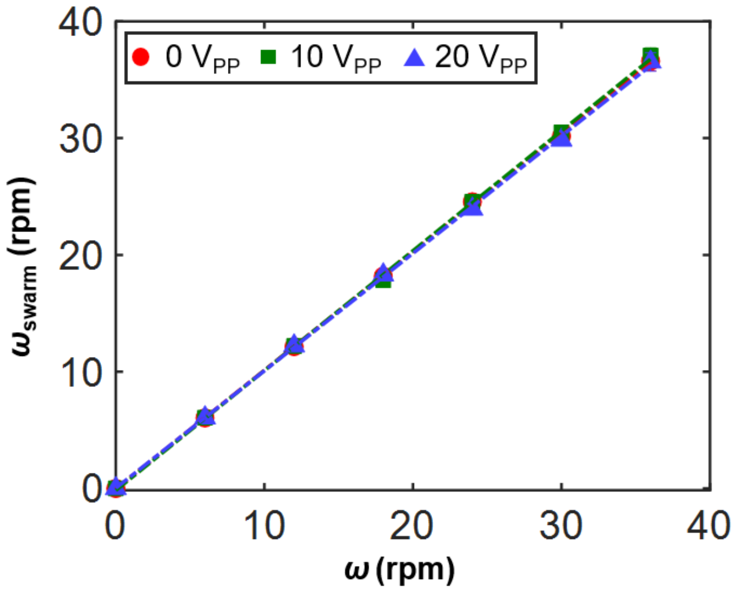

Supplementary Fig. 5. Swarm angular velocity versus the magnetic rotational velocity. With different acoustic voltages, all fitting curves have a slope of approximately one, which indicates that microchains precisely follow the rotational magnetic field during multiple rolling cycles and the swarm angular velocity is nearly not affected by the acoustic voltage. The magnetic intensity was 21 mT. Each data point represents the average angular velocity analysed from 3–5 microchains (Source Data). Error bars represent the standard deviation (s.d.) of data.

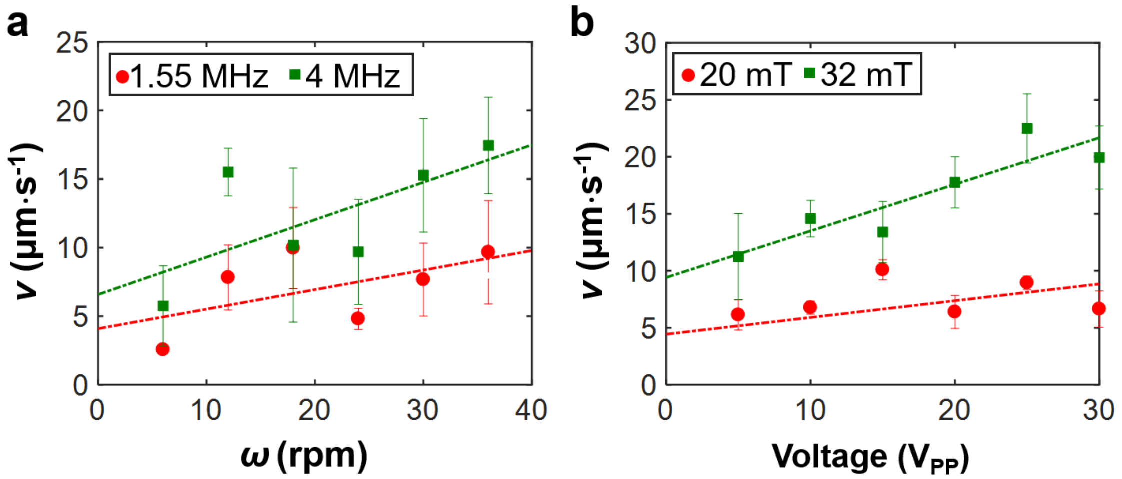

Supplementary Fig. 6. Characterizations of microchains rolling along the acoustic virtual wall in the open acoustic chamber. (**a**) The plot characterizes the translational velocity of microchains versus the magnetic rotational velocity at different acoustic excitation frequencies. The acoustic excitation voltage was 20 V_{PP} and the magnetic intensity was 21 mT. (**b**) The plot characterizes the translational velocity of microchains versus the acoustic excitation voltage at different magnetic intensities. The acoustic excitation frequency was 1.55 MHz and the magnetic rotational velocity was 18 rpm. The fittings were performed with the first-order linear function model, $y = ax+b$. These plots demonstrate that: (1) The translational velocity increases with the increasing acoustic frequency. These results are due to a higher excitation frequency can produce a higher ARF. Subsequently, the higher ARF can cause a larger off-center rotation to increase the translational velocity. (2) The translational velocity is also increased by increasing the intensity of the magnetic field due to the stronger dipole-dipole interactions make the self-assembly microswarms more stable by reducing the possibility to break into short chains during rolling. Each data point represents the average translational velocity analysed from 3–5 microchains (Source Data). Error bars represent the standard deviation (s.d.) of data. The spread of the data points can be explained as follows: (1) the data was captured from five different microchains from different acoustic pressure nodal lines, which exhibit a relatively large variety of lengths; (2) the dynamic acoustic pressure nodal line was not perfectly straight. Additionally, it is worth noting that the translational velocity of microchains in the capillary is bigger than that in the acoustic chamber, which can be explained as: (1) as the distance between two opposite transducers decreases, the intensity of acoustic field decays less; (2) the hydrodynamic interaction in the narrow capillary boundary may accelerate the translational velocity (which is beyond the scope of this study and will be explored in future work).

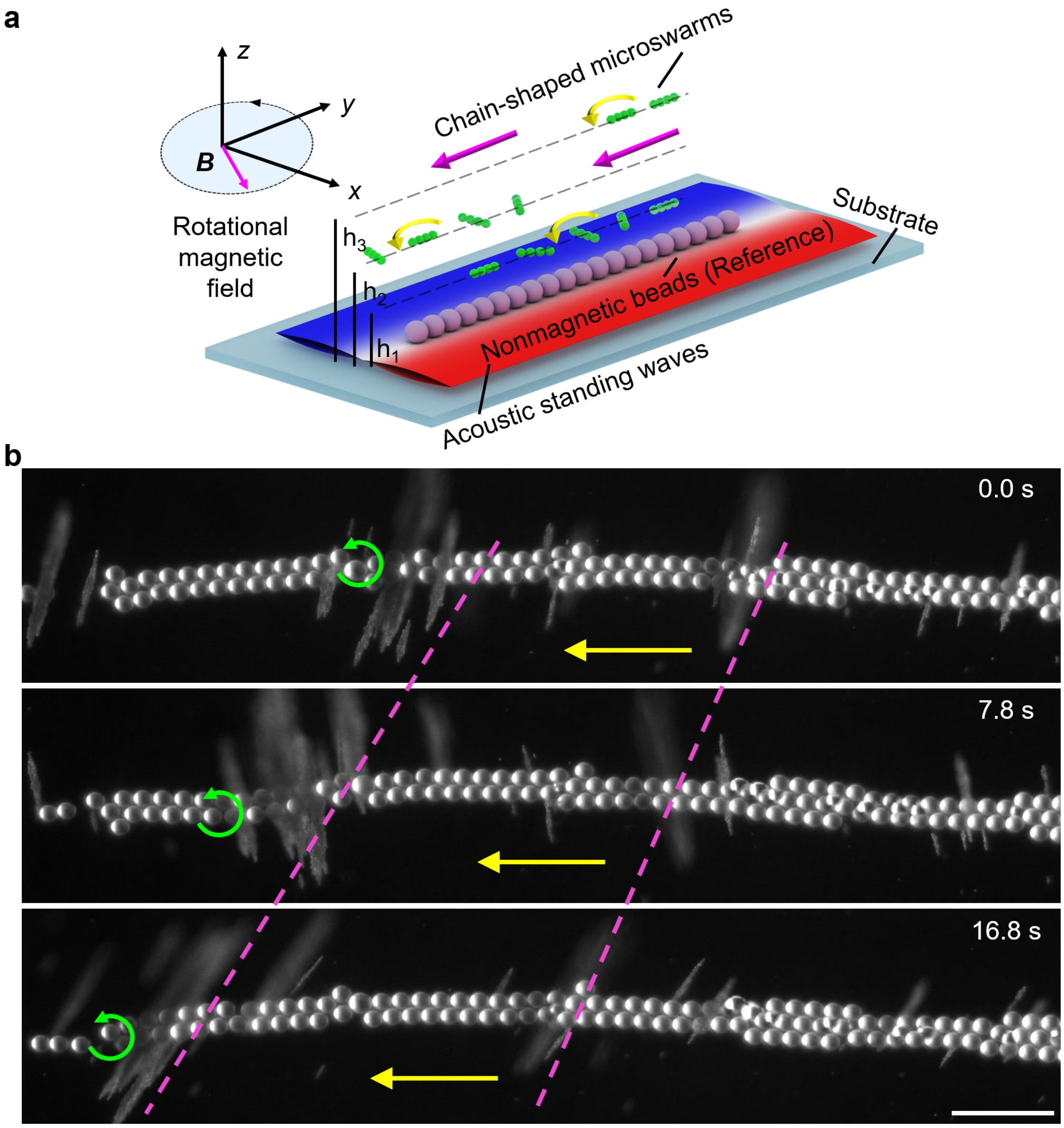

Supplementary Fig. 7. Microswarms rolling along the acoustic virtual wall at different suspended planes. (**a**) Schematic of chain-shaped microswarms rolling at different suspended planes. The yellow curved arrow and the pink arrow denote the magnetic rotational direction and the translational direction, respectively. (**b**) Control experiment demonstrates microchains synchronously performed rolling along the acoustic virtual wall at different suspended planes. In contrast, no noticeable motion was observed of the nonmagnetic 15 μm polystyrene microbead laying below. The fuzzier the particle, the farther away it was from the polystyrene bead layer. The green curve arrow, yellow arrow, and pink dotted line denote the magnetic rotational direction, net translational direction, and displacement, respectively. Scale bar, 100 μm. During experiments nonmagnetic polystyrene beads were injected into the liquid first. Due to their larger mass, the polystyrene microbeads sediment rapidly to the bottom substrate, allowing them to act as a reference point. Then, magnetic particles were injected into the acoustic chamber. Following, a 1D acoustic standing wave field was introduced in the chamber, generating acoustic pressure nodal lines at multiple suspended planes, i.e. across different heights along the z-axis. The two types of particles were trapped along those pressure nodal lines. The vertical component of the ARF keeps magnetic particles suspended in the liquid [5–8]. We estimated the respective distances between the substrate and magnetic microparticles trapped at various suspended planes as: ∼15 μm, ∼35 μm, ∼70 μm, ∼110 μm, ∼400 μm, and even ∼1200 μm. These heights were measured by tuning and calibrating the focus knob of the inverted microscope. Tuning the focus knob allows the viewer to change the z-position that the microscope is focused on. We first moved the objective so that the 15 μm polystyrene microbeads on the glass substrate was in focus and marked the vertical position as Z_0. Then we moved the objective to bring the suspended planes into focus in sequence and marked their respective vertical positions as Z_1, Z_2, Z_3 .... Finally, by subtraction ($H = Z_i - Z_0$), we obtained the height of each suspended plane.

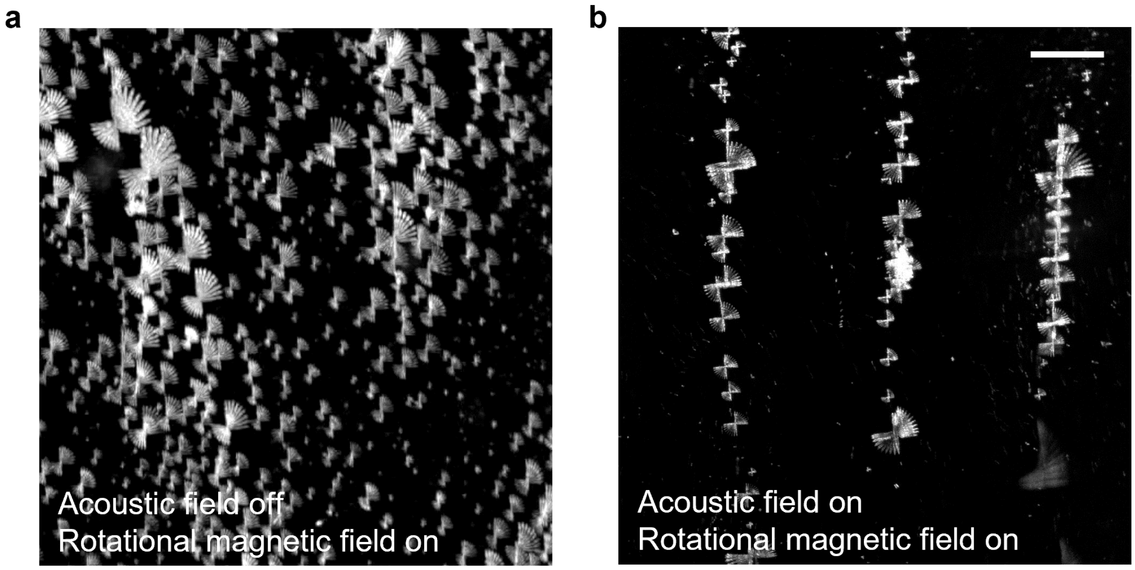

Supplementary Fig. 8. Superimposed time-lapse images of field control experiments. (**a**) Behavior of microchains in the rotational magnetic field without the acoustic field. Microchains arbitrarily disperse in the bulk liquid. Microchains show the off-center rotation *in situ* and no noticeable motion is observed. (**b**) Behavior of microchains in the rotational magnetic field with the one-dimensional acoustic standing wave field. Microchains show the off-center rotational along the acoustic pressure nodal lines and move forward. The magnetic rotational velocity was 18 rpm. The acoustic excitation voltage and frequency were 20 V_{PP} and 1.55 MHz, respectively. Scale bar, 100 μm.

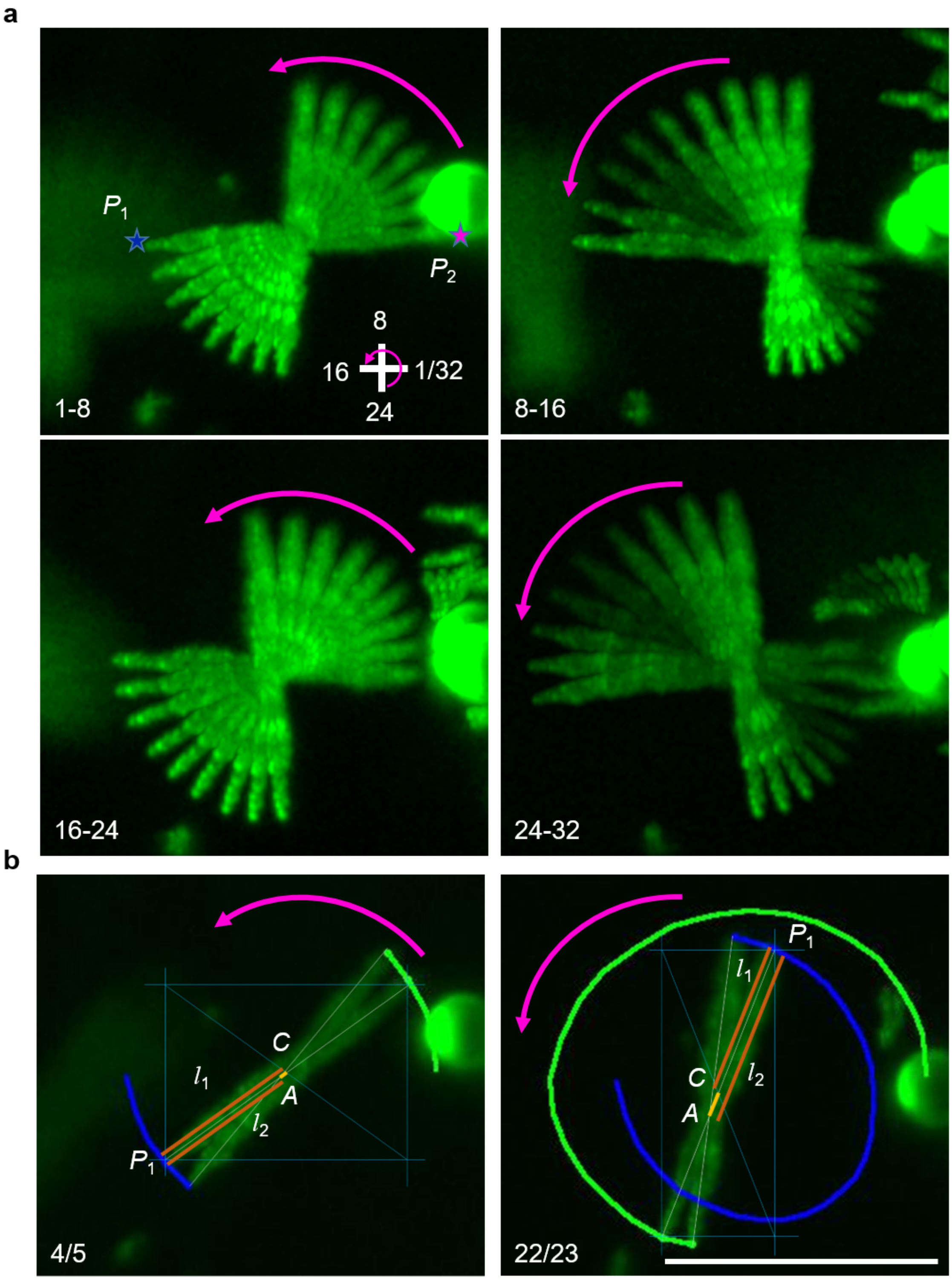

Supplementary Fig. 9. Superimposed time-lapse images of a single microschain in a rotational cycle. (**a**) The angular spacing decreases in the farm range 1-8 and 16-24. On the contrary, it increases in the range 8-16 and 24-32. The frame has the same time interval, 0.1587 s, from 1 to 32. The pink curved arrow denotes the counterclockwise rotational direction. The number refers to the overlapped frames. The acoustic excitation voltage and frequency were 20 V_{PP} and 1.55 MHz, respectively. The magnetic rotational direction was counterclockwise, and the magnetic rotational velocity and intensity were 12 rpm and 21 mT, respectively. (**b**) Measurement method of the distance between rotational center and geometry center. The distance P_1C is defined as l_1 and the distance P_1A is defined as l_2 (denoted by the orange lines). Thus, the off-center distance $l(t) = l_2$ - l_1 (denoted by the yellow line). When $l_2 < l_1$, the sign of $l(t)$ is defined as minus; When $l_2 > l_1$, the sign of $l(t)$ is defined as plus. The white line denotes the rotating microchain and the light blue lines are the auxiliary positioning lines. Scale bar, 50 μm.

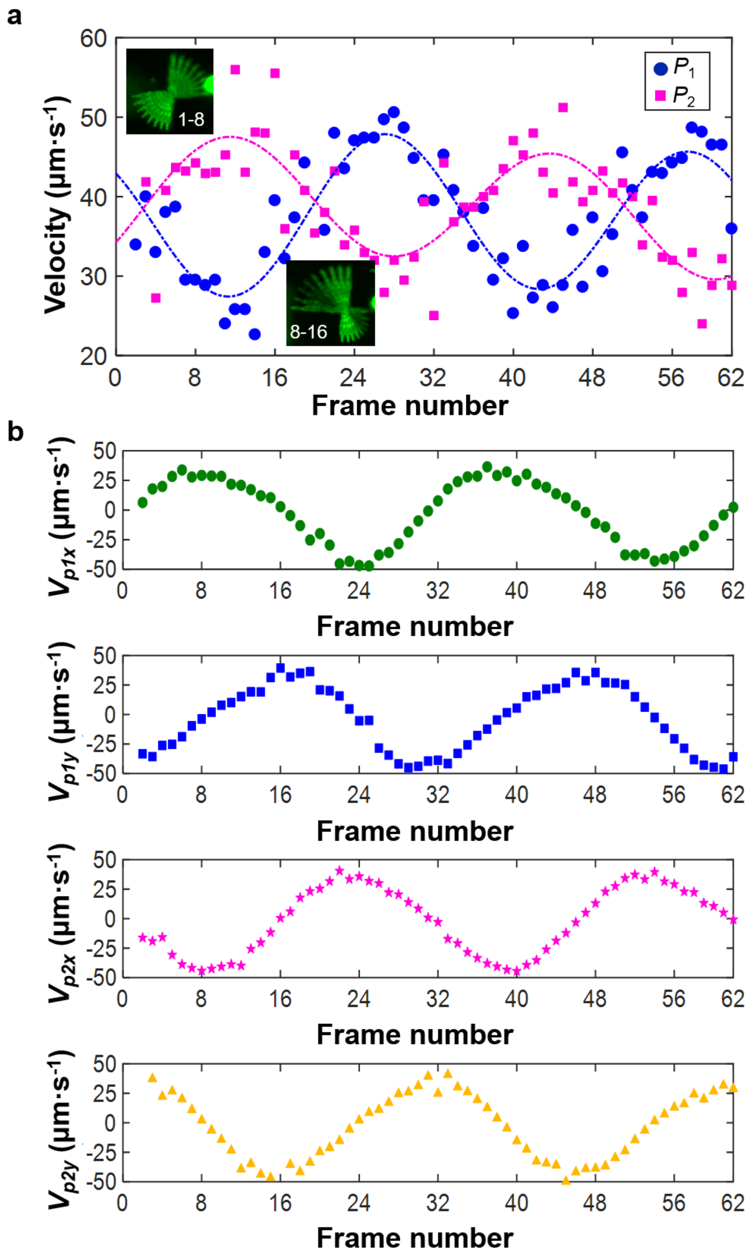

Supplementary Fig. 10. Tracked velocities of the microchain's two endpoints (P_1 and P_2) against the rolling frames (**a**) Linear velocities (Source Data). (**b**) Velocity components in the x and y directions, respectively (Source Data).

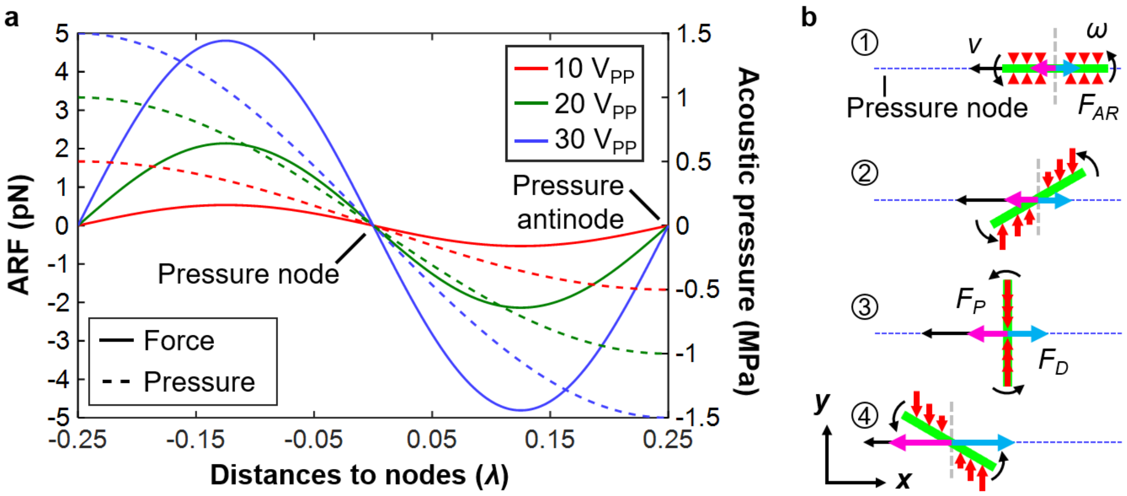

Supplementary Fig. 11. Acoustic radiation force estimation and analysis. (**a**) Acoustic radiation force and acoustic wave pressure estimation of a single magnetic particle versus the distance to the acoustic pressure node. The ARF shows sinusoidal dependence on the particle's position in the acoustic standing wave field, with maximum ARF being achieved when the particle is sited at one-eighth of the acoustic wavelength from the pressure node. As the particle moves towards the pressure node, the ARF decreases to zero (Source Data). The acoustic excitation frequency was 1.55 MHz. (**b**) Acoustic radiation force analysis of the microchain at different orientations. over one cycle of rolling, the ARF acting on the microchain always points to the pressure nodal line, but its magnitude varies as the microchain rotates. When the microchain aligns along the pressure nodal line (phase 1), it experiences the minimum ARF. Then, as it rotates away from the pressure nodal line in the counterclockwise direction, the ARF increases gradually. In phase 2, the ARF tends to impede the microchain's rotation, as the acoustic radiation torque opposes the magnetic torque. When the microchain is perpendicular to the pressure nodal line (phase 3), it experiences the maximum ARF and the ARF gradually decreases, as it rotates towards the pressure nodal line. In phase 4, the ARF tends to accelerate the rotation of the microchain on account of the acoustic radiation torque having a similar direction to the magnetic torque. The blue dotted line denotes the acoustic pressure nodal line. The red arrow, pink arrow, wathet arrow, and black curved arrows denote the acoustic radiation force, propulsion force, drag force, and magnetic torque, respectively. The black arrow along the pressure nodal line denotes the translational direction.

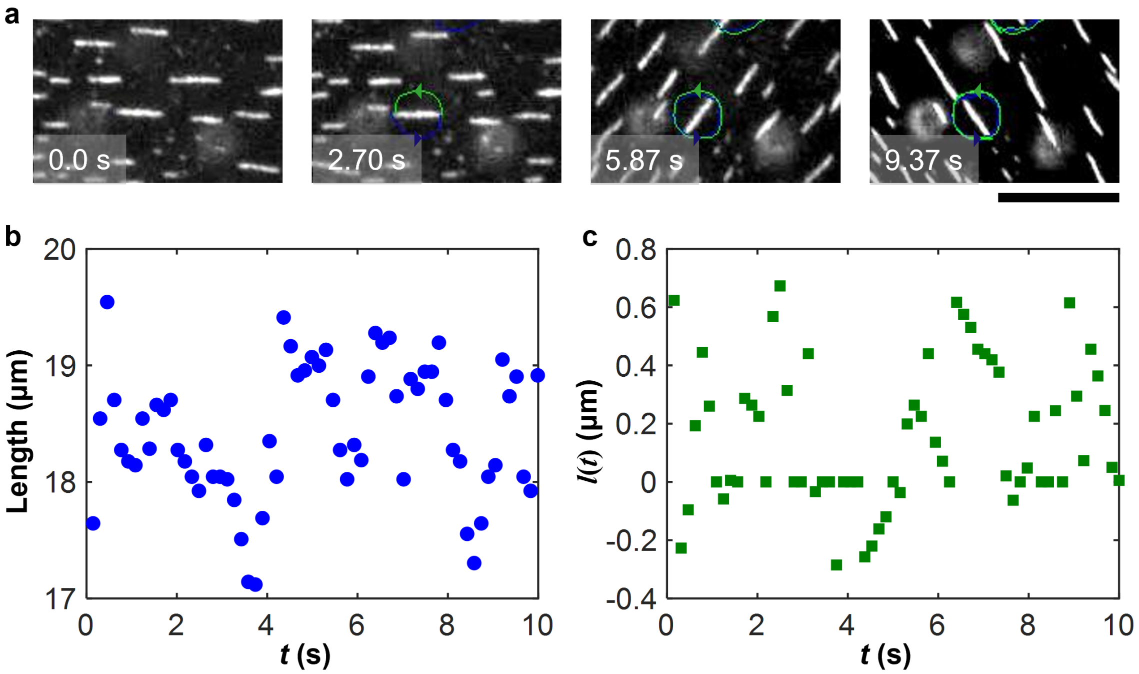

Supplementary Fig. 12. Control experiment of single microchain tracking and analysis. (**a**) Image sequence of a microchain undergoing counterclockwise rotation in the rotational magnetic field in the absence of the acoustic field. The magnetic rotational velocity and intensity were 12 rpm and 21 mT, respectively. Scale bar, 50 . (**b**) Length of the microchain calculated based on the coordinates of the two endpoints (Source Data). (**c**) Distance between the geometric and rotational centers of the microchain against the rotational time, where one rotational cycle was 5 seconds (Source Data). $l(t)$ does not show a sinusoidal change. The magnitude difference is slight and is from manual tracking error.

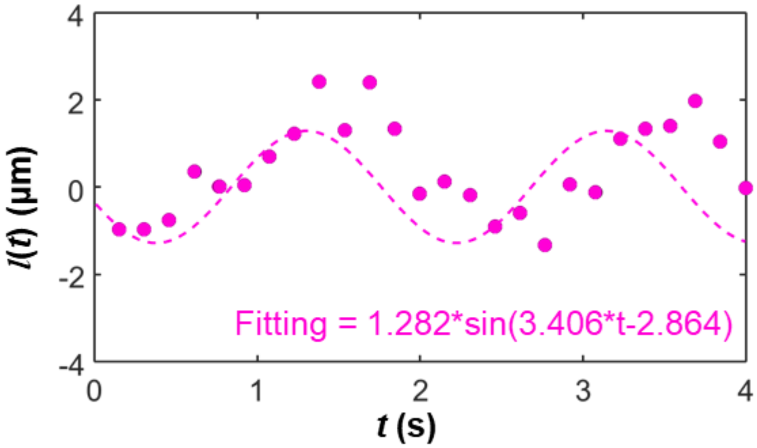

Supplementary Fig. 13. Variable distance between the geometric and rotational centers of the microchain against rotational time. The length of the rolling microchain was 15.6 μm. The acoustic excitation voltage and frequency were 20 V_{PP} and 1.55 MHz, respectively. The magnetic rotational direction was clockwise, and the magnetic rotational velocity and intensity were 30 rpm and 21 mT, respectively (Source Data). The variable $l(t)$ was fitted as $l(t) = 1.282 * (3.406 * t - 2.864)$. The values of the time-averaged displacement δ and velocity v of the tracked microchain in one rotational cycle read $\delta_{theory} = 4.028$ μm and $v_{theory} = 2.014$ $\mu m \cdot s^{-1}$ which are consistent with the tracked experimental data of $\delta_{exp} = 4.095$ μm and $v_{exp} = 2.047$ $\mu m \cdot s^{-1}$. The slight difference came from the manual tracking error. Since different microchains will have different $l(t)$, with suitable fitting, we believe the model can predict microchain rolling behaviours in variable experimental conditions.

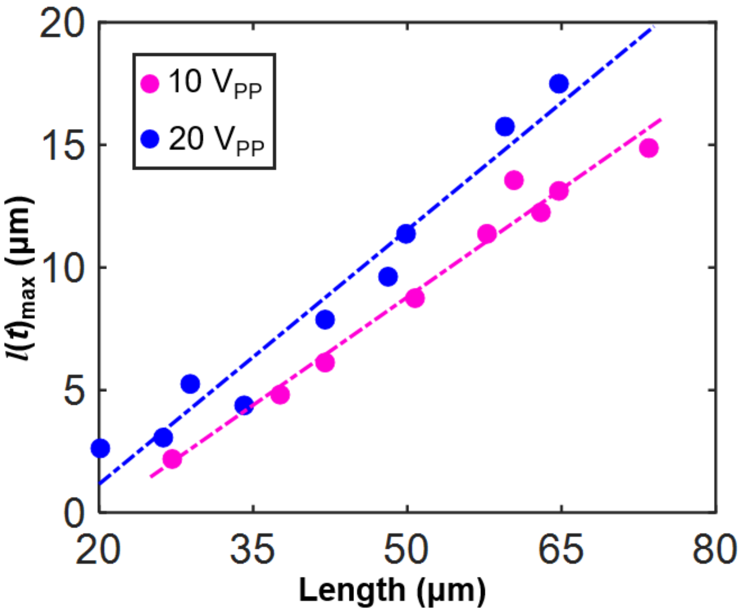

Supplementary Fig. 14. The maximum of the off-center distance versus the length of microchains at different acoustic excitation voltages. $l(t)_{max}$ is approximately equal to b_1 in the model. With a constant excitation voltage, the maximum increases with the swarm length; and as the excitation voltage increases, the maximum also increases. These fittings were performed with the first-order linear function model, $y = ax + b$. The magnetic rotational velocity and intensity were 30 rpm and 21 mT, respectively (Source Data).

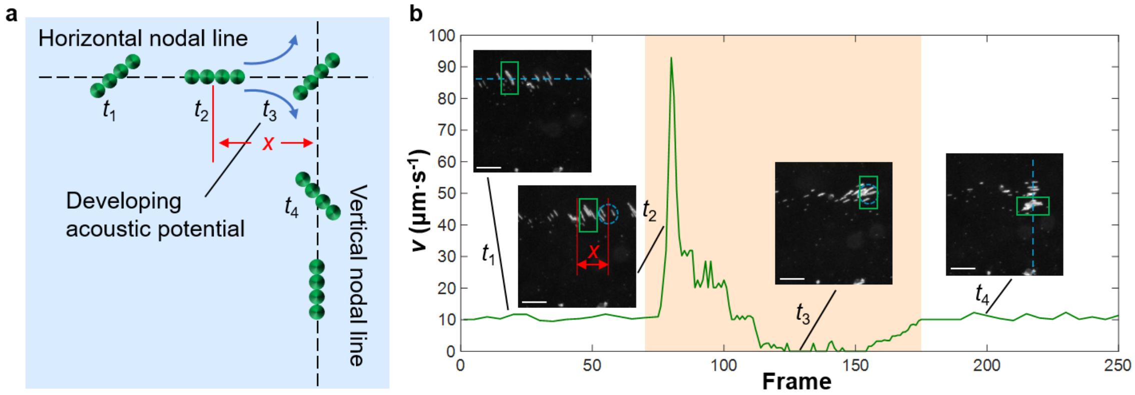

Supplementary Fig. 15. Dynamic process of the reorientation of the acoustic virtual wall. (**a**) Schematic illustrating reorientation of the acoustic virtual wall. At t_1, the microchain rolls along the horizontal acoustic pressure nodal line; at t_2, it rolls close to the center point of the 2D pressure node with distance x; at t_3, the microchain is accelerated and pushed along an arbitrary trajectory toward the pressure node because of the redeveloped acoustic potential; as the distance x decreases, the microchain slows down and only rotates at the pressure node; at t_4, the microchain rolls along the vertical acoustic pressure nodal line. (**b**) Plot shows the translational velocity of a tracked microchain at different time frames. During the development of the pressure node, the microchain is accelerated first and then slows down to 0 due to the reducing radiation force. The microchain has a stable translational velocity when rolling along the horizontal and vertical pressure nodal line at ~10.63 $\mu m \cdot s^{-1}$. The acoustic excitation voltage and frequency were 20 V_{PP} and 1.55 MHz, respectively. The magnetic rotational direction was clockwise, and the magnetic rotational velocity and intensity were 24 rpm and 21 mT, respectively (Source Data). Insets show the movements of the tracked microchain at different rolling time points. The switching operation is distinguished from the rolling motion, and the resulting motion is highly related to the operating voltage and speed. The green box shows the tracked microchain. The blue dotted line and the dotted circle denote the acoustic pressure nodal line and the pressure node, respectively. Scale bar, 50 μm.

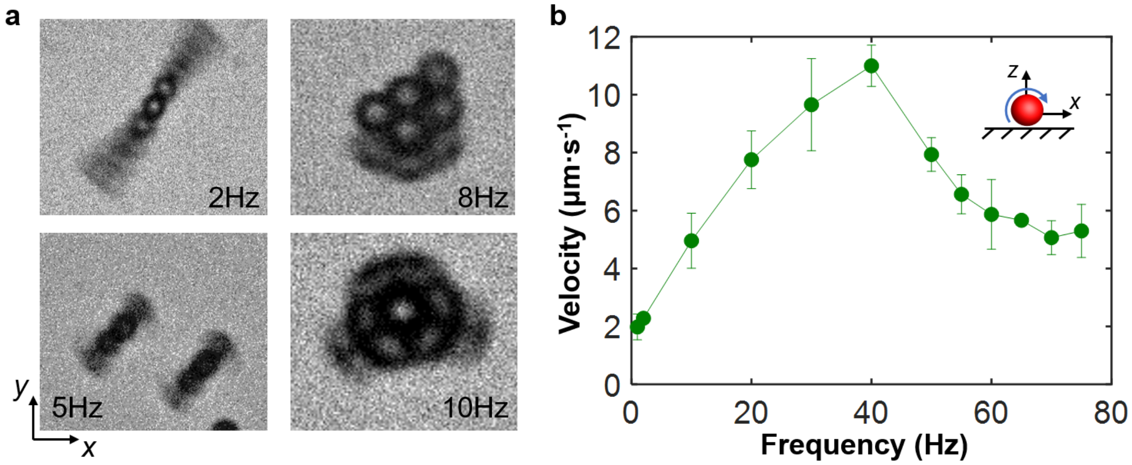

Supplementary Fig. 16. Step-out frequencies of the microswarm and a single particle. (**a**) Variable self-assembly shapes of magnetic particles with different magnetic rotational frequencies in the $x - y$ rotational magnetic field of 21 mT. (**b**) Rolling velocity of a single particle versus the magnetic rotational frequency within the $x - z$ rotational magnetic field of 21 mT. The inset schematic shows the experimental rolling motion. Within the 21 mT rotational magnetic field, $\mathbf{B}(t) = B_0 \cos\omega t \mathbf{e}_x + B_0 \sin\omega t \mathbf{e}_z$ (the rotational axis is y-axis), the translational velocity of such a single particle increases almost linearly with the frequency till 40 Hz after which it decreases upon further increasing the frequency. Thus, the step-out frequency of the used single particle is around 40 Hz. Each data point represents the average translational velocity analysed from 3–5 microparticles (Source Data). Error bars represent the standard deviation (s.d.) of data.

Supplementary Tables

Supplementary Table 1. Verification of the rolling model with microchains under different experimental conditions.

Experimental conditions	Chain length (μm)	Rotational speed (rpm)	Maximum value of the off-center distance (μm)	Experimental displacement δ_{exp} (μm)	Experimental velocity v_{exp} (μm·s^{-1})	Theoretical displacement δ_{th} (μm)	Theoretical velocity v_{th} (μm·s^{-1})
In the capillary (2.02MHz, 10Vpp, 15 mT)	54.25	30	10.56	36.75	18.38	34.36	17.18
	38.50	30	5.88	17.61	8.80	17.87	8.93
	36.75	30	4.81	14.00	7.00	15.12	7.56
In the chamber (1.55MHz, 10Vpp, 21 mT)	49.60	30	4.00	12.00	6.00	12.57	6.28
In the chamber (1.55MHz, 20Vpp, 21 mT)	54.22	12	7.20	22.10	4.40	22.49	4.49
	15.60	30	1.30	4.10	2.05	4.03	2.01

Supplementary References

1. Yosioka, K. & Kawasima, Y. Acoustic radiation pressure on a compressible sphere. *Acustica* **5**, 167–173 (1955).
2. Fine, R. A. & Millero, F. J. Compressibility of water as a function of temperature and pressure. *The J. Chem. Phys.* **59**, 5529–5536 (1973).
3. Singh, F., Avasthi, D., Angelov, O., Berthet, P. & Pivin, J. Changes in volume fraction and magnetostriction of iron nanoparticles in silica under swift heavy ion irradiation. *Nucl. Inst. Methods Phys. B* **245**, 214–218 (2006).
4. Bridgman, P. W. *The compressibility of thirty metals as a function of pressure and temperature* (Harvard Univ. Press, Cambridge, 2013).
5. Petersson, F., Nilsson, A., Holm, C., Jönsson, H. & Laurell, T. Separation of lipids from blood utilizing ultrasonic standing waves in microfluidic channels. *Analyst* **129**, 938–943 (2004).
6. Shi, J., Huang, H., Stratton, Z., Huang, Y. & Huang, T. J. Continuous particle separation in a microfluidic channel via standing surface acoustic waves (ssaw). *Lab Chip* **9**, 3354–3359 (2009).
7. Wu, M. *et al.* Acoustofluidic separation of cells and particles. *Microsyst. Nanoeng.* **5**, 1–18 (2019).
8. Shi, J. *et al.* Three-dimensional continuous particle focusing in a microfluidic channel via standing surface acoustic waves (ssaw). *Lab Chip* **11**, 2319–2324 (2011).
