# Supplementary material for: Rolling microswarms along acoustic virtual walls
Source: Nat Commun. 2022 Nov 29;13:7347. doi: 10.1038/s41467-022-35078-8 (PMC9708833; doi:10.1038/s41467-022-35078-8)
Supplement: Supplementary file 2 — Description of Additional Supplementary Files [file 41467_2022_35078_MOESM2_ESM.pdf]

Description of Additional Supplementary Files

Rolling Microswarms along Acoustic Virtual Walls

Zhiyuan Zhang, Alexander Sukhov, Jens Harting, Paolo Malgaretti, and Daniel Ahmed

Corresponding Author: Daniel Ahmed

E-mail: dahmed@ethz.ch

This PDF file includes:

Legends for Supplementary Movies 1 to 6

Other supplementary materials for this manuscript include the following:

Supplementary Movies 1 to 6

Supplementary Movie 1. **Microswarms roll along the acoustic virtual wall**. The acoustic excitation voltage and frequency were 20 V_{PP} and 1.55 MHz, respectively. The magnetic rotational direction was clockwise, and the magnetic rotational velocity and intensity were 18 rpm and 21 mT, respectively.

Supplementary Movie 2. **Dynamic orientation switching of the acoustic standing wave field**. Amplitude modulation: when the excitation voltage of the transducer pair was $A : B = 20 : 20$ V_{PP}, the acoustic pressure node tended to be the pressure nodal point; when the excitation voltage of the transducer pair was $A : B = 20 : 1$ V_{PP}, the acoustic pressure node tended to be the horizontal pressure nodal line. When the excitation voltage of the transducer pair was $A : B = 1 : 20$ V_{PP}, the acoustic pressure node tended to be the vertical pressure nodal line. The acoustic excitation frequency was kept at 1.55 MHz.

Supplementary Movie 3. **Microswarms exhibit bidirectional rolling**. Microswarms executed left-to-right rolling in a clockwise rotational magnetic field, followed by right-to-left rolling upon the rotational direction of the magnetic field was switched to be counterclockwise. Then, microswarms executed bottom-to-top rolling in a counterclockwise rotational magnetic field, followed by top-to-bottom rolling upon the rotational direction of the magnetic field was switched to be clockwise. The acoustic excitation voltage and frequency were 20 V_{PP} and 1.55 MHz, respectively. The magnetic rotational velocity of both demonstrations was 24 rpm and 30 rpm, respectively. The magnetic intensity was 21 mT.

Supplementary Movie 4. **Microswarms synchronously roll at different suspended planes.** The acoustic excitation voltage and frequency were 20 V_{PP} and 1.55 MHz, respectively. The magnetic rotational direction was counterclockwise, and the magnetic rotational velocity and intensity were 12 rpm and 21 mT, respectively.

Supplementary Movie 5. **Trajectory tracking of a single microchain in one rolling cycle**. The acoustic excitation voltage and frequency were 20 V_{PP} and 1.55 MHz, respectively. The magnetic rotational direction was counterclockwise, and the magnetic rotational velocity and intensity were 12 rpm and 21 mT, respectively.

Supplementary Movie 6. **Microswarms write the word "*ETH*"**. The trajectory of microswarms was controlled by dynamically switching the orientation of the virtual wall and the rotational direction of the magnetic field.
